# Refractive Index and Strain Modulation Tailor the Afterglow of Nanocomposite Films

**DOI:** 10.1021/acs.jpclett.5c02216

**Published:** 2025-10-23

**Authors:** Victor Castaing, Manuel Romero, Théophile Drion, Alberto J. Fernández-Carrión, Gabriel Lozano, Hernán Míguez

**Affiliations:** Institute of Materials Science of Seville, 16778 Spanish National Research Council − University of Seville, C/Américo Vespucio 49, 41092 Seville, Spain

## Abstract

Tailoring the unique delayed and long-lasting luminescence of persistent phosphors is crucial for their application in anticounterfeiting, data storage, imaging displays, and AC-driven lighting. We introduce a novel strategy to achieve this by modifying the refractive index of persistent phosphor transparent coatings. Specifically, we developed ZnGa_2_O_4_:Cr^3+^/SiO_2_ nanocomposite films with tunable refractive indices from 1.45 to 1.7. This tunability allowed us to precisely control the Cr^3+^ radiative decay rate, resulting in a substantial 1.7-fold increase in both luminescence and afterglow brightness. Furthermore, our approach uniquely influences the intrinsic charging rate of the phosphor, a mechanism attributed to the strain induced on the ZnGa_2_O_4_:Cr^3+^ nanocrystals by the presence of SiO_2_. This work demonstrates an unprecedent ability to manipulate the afterglow kinetics without altering the material composition, opening new avenues for designing and optimizing persistent luminescence materials.

## Graphical abstract

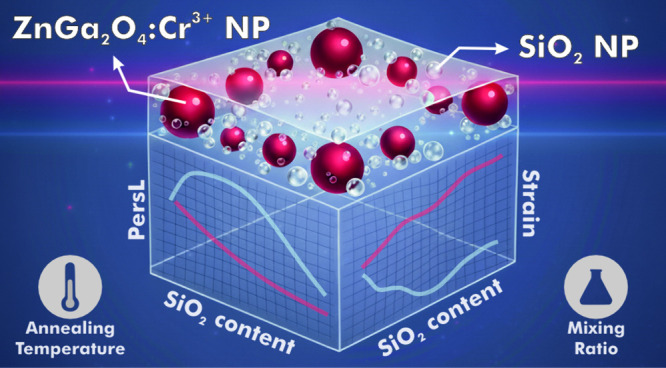

Persistent luminescence (PersL) phosphors, characterized by their unique ability to absorb and then gradually re-emit light over extended periods, offer transformative potential across diverse fields, including bioimaging, security, and optoelectronic displays.^1,2^ This remarkable phenomenon arises from the interplay of emitting centers (typically rare-earth or transition-metal ions) and trapping centers (e.g., vacancies, antisites or codoping ions) that store part of the absorbed optical energy and release it over time, resulting in afterglow.^1,2^ Although compositional engineering remains the primary route to tailor afterglow properties like color and duration,^3–8^ emerging evidence highlights the critical, yet underexplored, influence of the phosphor’s local environment, particularly on afterglow kinetics.^9–12^ Given that PersL involves intricate optical and thermal processes, including photon absorption, charge trapping, thermal and optical release, and emission,^13–15^ altering the surrounding optical or thermal environment offers a powerful, largely untapped pathway to manipulate their trapping capacities and, consequently, their afterglow characteristics.^11,16^ Despite the widespread success of photonic design strategies in optimizing other light-emitting materials,^17–20^ their application to engineer PersL remains an exciting and largely unseized opportunity.

Addressing this gap, we herein investigate the fundamental role of refractive index modification as the simplest route to tune the optical environment of PersL phosphors.^21^ We specifically focus on ZnGa_2_O_4_:Cr^3+^ (ZGO:Cr) nanocrystal-based transparent coatings, fabricating nanocomposite films with varying SiO_2_ content to precisely control the effective refractive (*n*_eff_) index of the films from 1.45 to 1.7. This strategy allows for a systematic study of its impact on PersL characteristics. Our work reveals that modulating the refractive index directly influences the Cr^3+^ decay rate, leading to significant enhancements in both luminescence and afterglow brightness. Crucially, through detailed analysis of luminescence kinetics and structural properties, we uncover an unprecedented secondary mechanism: the SiO_2_ matrix induces mechanical strain on the embedded ZGO:Cr nanoscrystals, which, alters their intrinsic charge trapping and release kinetics.^15^ This dual influence, optical through refractive index, and mechanical through strain, enables a powerful new paradigm for tailoring PersL. Our findings establish a versatile, noncompositional methodology for engineering the optical and afterglow properties of transparent thin films, opening new avenues for advanced photonic materials.

To investigate the influence of the *n*_eff_ on PersL properties, transparent coatings were fabricated by blending ZGO:Cr PersL nanoparticles (PersLNPs, *n* = 1.94) with SiO_2_ nanoparticles (*n* = 1.46). The particle ratio of ZGO:Cr to SiO_2_ (Z:S) was systematically varied from 100:0, i.e., pure ZGO:Cr, to 10:90. These mixtures were then processed into thin films on fused silica substrates via spin coating (see Figure 1a).

**Figure 1:**
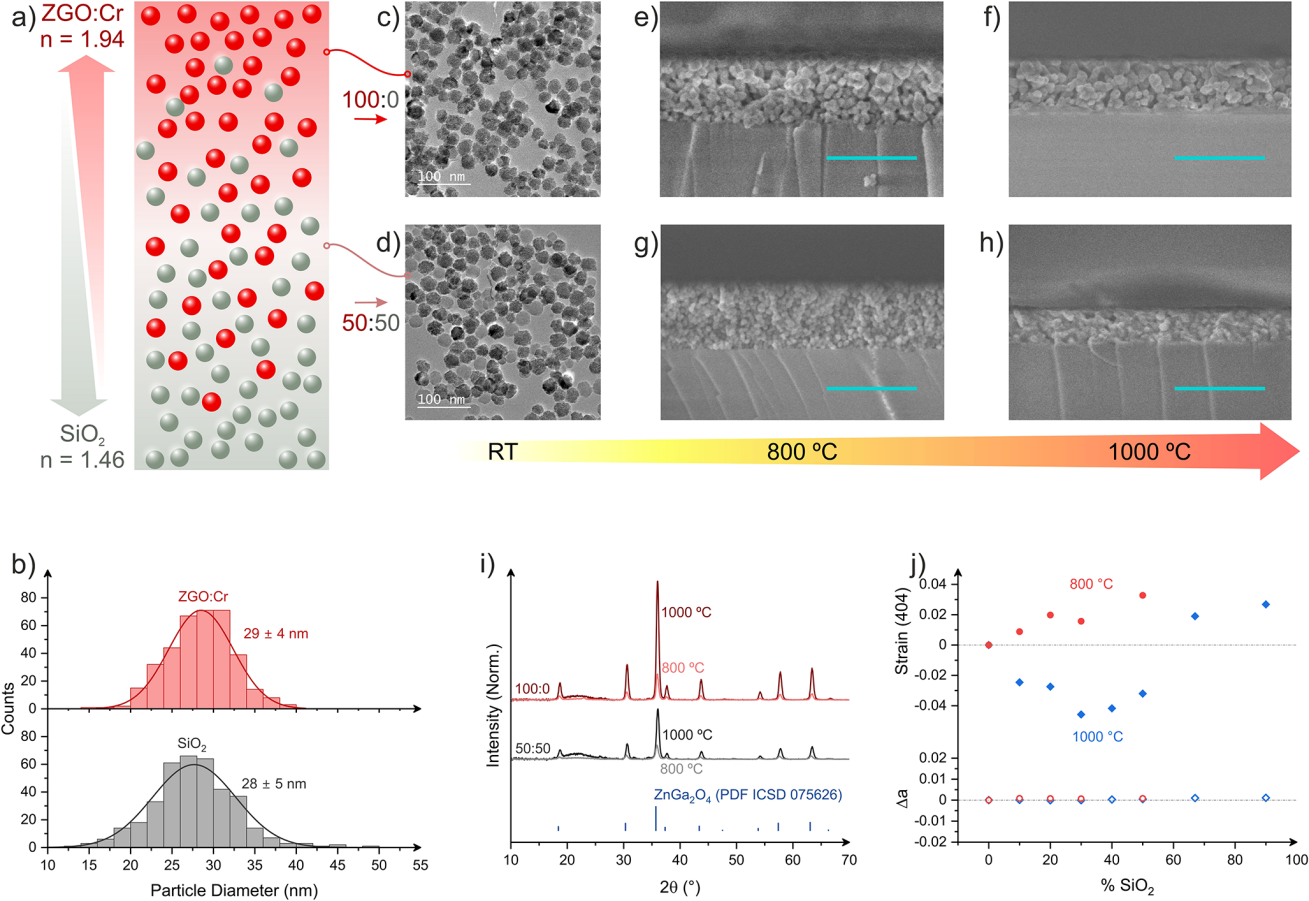
(a) Schematic of the strategy used to tune the refractive index of layers. (b) The size distribution of the particles was estimated by measuring the diameter of more than 200 particles using transmission electron microscopy (TEM) images. TEM images of representative particle mixtures: (c) 100:0 and (d) 50:50. SEM pictures of a cross section of different films: (e) 100:0 film processed at 800 °C, (f) 100:0 film processed at 1000 °C, (g) 50:50 film processed at 800 °C, and (h) 50:50 film processed at 1000 °C. (i) XRD diagrams of the 100:0 and 50:50 films that were processed at 800 and 1000 °C. The ZGO lines extracted from the reference pattern PDF ICSD 075626 are shown below. (j) Strain along the (404) crystallographic plane and variation in the cubic lattice parameter of samples annealed at 800 °C (red) and 1000 °C (blue), as a function of the SiO_2_ content.

ZGO:Cr PersLNPs were synthesized following a microwave-assisted hydrothermal route.^22^ To ensure optimal processing of the nanocomposite films, the synthesis parameters were adjusted to produce ZGO:Cr with an average diameter of 29 ± 4 nm. This closely matches the size of commercial SiO_2_ NPs (28 ± 5 nm), as shown in Figures 1b and 1d, which favors the preparation of uniform nanocomposite films. Notice that mixing nanoparticles of significantly different sizes may lead to agglomeration during film formation, resulting in inhomogeneous coatings of poor optical quality.

Films were subsequently annealed at two distinct temperatures: 800 and 1000 °C. Both annealing conditions consistently produced homogeneous and transparent layers (see Figures 1e and 1f, as well as ). While the same suspension concentration was used for all coatings, both the processing temperature and the Z:S ratio influence the resultant film thickness and morphology. For example, pure ZGO:Cr layers calcined at 800 °C yielded film approximately 370 nm thick with notable porosity and low degree of sintering (Figure 1e). Increasing the annealing temperature to 1000 °C induced significant particle sintering and coalescence, accompanied by pores shrinkage and layer densification, resulting in a reduced thickness of around 310 nm, as shown in Figure 1f.

The presence of SiO_2_ NPs exerted a distinct influence on film morphology and sintering behavior. At 800 °C, SiO_2_ effectively prevented ZGO:Cr particle sintering, maintaining discrete particles of approximately 30 nm and preserving film thickness, e.g., 380 nm for a 50:50 Z:S ratio (see Figure 1g). However, annealing SiO_2_-containing films at 1000 °C lead to substantial film thickness reduction, e.g., 200 nm for the 50:50 Z:S ratio (Figure 1h), and the apparent elimination of porosity. These observations can be attributed to the melting of the silica nanoparticles (SiO_2_ NPs, LUDOX) at this processing temperature, which is favored by the presence of sodium (Na) on the surface of the commercial SiO_2_ NPs. Additionally, to rule out the possibility of SiO_2_ sublimation, we conducted a control experiment in which a thick film composed solely of SiO_2_ nanoparticles was annealed at 1000 °C under the same conditions used for the nanocomposites. SEM images clearly reveal the presence of a continuous SiO_2_ layer after annealing (see ).

## Structural Characterization and Strain Analysis

All synthesized samples exhibited the characteristic spinel phase of ZGO (PDF ICSD 075626; see Figure 1i, as well as ). As expected, decreasing the Z:S ratio led to a reduction in the intensity of ZGO diffraction peaks, relative to the broad amorphous band at 2θ = 21 °, which is associated with amorphous SiO_2_. Strain (ε) was quantified from Rietveld refinements of the (404) reflection using ε = (*d* – *d*_0_)/*d*_0_, where *d* corresponds to the interplanar spacing and *d*_0_ corresponds to the interplanar spacing of the silica-free reference. This analysis revealed a significant strain influence on the ZGO:Cr nanoscrystals induced by the SiO_2_. For films annealed at 1000 °C, the introduction of SiO_2_ caused a negative strain (compression) in the nanocrystals compared to pure ZGO:Cr films, reaching up to −0.035 for 30% SiO_2_ content, while the cubic lattice parameter remained constant (Figure 1j). Conversely, in nanocomposites primarily made of silica (e.g., 90% SiO_2_), the nanocrystals experienced positive strains (tension) up to 0.027, likely due to the formation of cracks that release local compression (see ). In contrast, for films annealed at 800 °C, where SiO_2_ does not melt but acts as a physical spacer, the ZGO:Cr nanocrystals experienced a growing tensile strain with increasing SiO_2_ content. This suggests that at 800 °C, SiO_2_ primarily limits the necking and sintering of ZGO:Cr NPs, rather than inducing compressive forces from melting and shrinkage.

## Effective Refractive Index Analysis

The *n*_eff_ of the calcined nanocomposite layers was determined by fitting ellipsometry spectra or reflectance and transmittance spectra, measured using a UV–vis–NIR spectrophotometer coupled with a double goniometer (). For the pure ZGO:Cr reference layer calcined at 1000 °C, a *n*_eff_ of 1.60 was observed, which is notably lower than the refractive index of bulk ZGO (*n*_ZGO_ = 1.94).^23^ This reduction is primarily attributed to the inherent porosity of the layer, estimated to be approximately ∼32% from ellipsometry fittings (where *n*_air_ = 1). The pure ZGO:Cr layer calcined at 800 °C exhibited a similar *n*_eff_ of 1.58, with the slight decrease likely correlated with the reduced sintering at this lower temperature. The *n*_eff_ of layers calcined at 800 °C progressively decreases with increasing SiO_2_ content (Figure 2a). This is due to the lower refractive index of SiO_2_ (*n*_SiO_2__ = 1.46) compared to *n*_ZGO_, and the fact that porosity remains constant within the series. Detailed refractive index values and the proportions of ZGO:Cr, SiO_2_ and air from the best fits are provided in .

**Figure 2:**
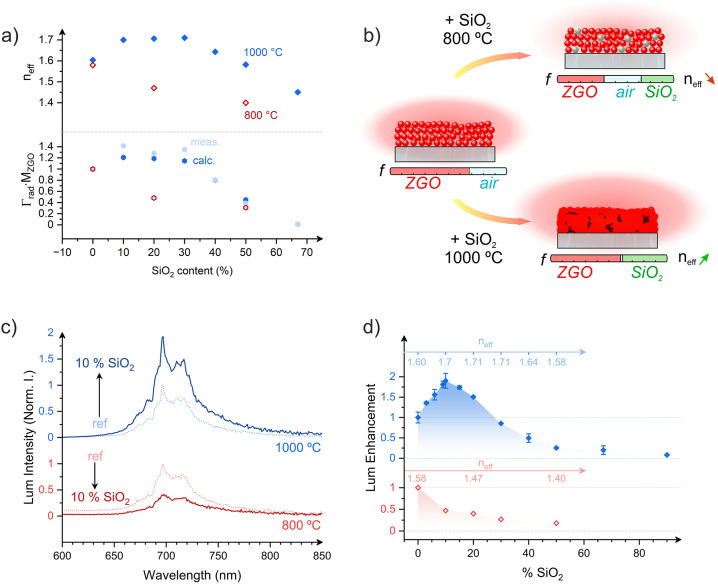
a) Refractive index of layers as a function of the Z:S ratio estimated from ellipsometry fits. Below, the calculated Γ_rad_·*M*_ZGO_ is represented as a function of the Z:S ratio. (b) Schematics of the composition fraction of films with different processing conditions. (c) Lum spectra of 100:0 and 90:10 layers calcined at 800 °C (red) and 1000 °C (blue) excited in the UV range (330 nm). (d) Lum enhancement of layers calcined at 800 °C (red) and 1000 °C (blue), represented as a function of the Z:S ratio with excitation in the UV range (330 nm).

Interestingly, films calcined at 1000 °C demonstrated an unconventional increase in *n*_eff_, reaching a maximum of 1.71 for a 70:30 (Z:S) particle ratio, before decreasing with further SiO_2_ addition (Figure 2a). As discussed above, this behavior is attributed to the melting of SiO_2_ NPs at 1000 °C, facilitating the filling of the voids of the coating and promoting densification. Evidence of SiO_2_ melting and subsequent densification was further supported by SEM images of pure SiO_2_ coatings calcined at 1000 °C (). The porosity of the films was estimated using the Bruggeman effective medium approximation using the estimated *n*_eff_ values and the known refractive indices of ZGO, SiO_2_, and air (see ). This analysis confirms the densification, revealing a significantly reduced porosity between 0 and 8% for films calcined at 1000 °C containing SiO_2_. Therefore, the combined manipulation of particle ratio and annealing conditions allows for a precise and broad adjustment of the *n*_eff_, spanning from 1.40 and 1.71 (see Figure 2b). In the subsequent sections, we analyze and discuss the direct effects of this controlled refractive index variation on the optical properties of the PersL coatings.

## Luminescence Intensity Analysis

Pure ZGO:Cr coatings, calcined at both 800 and 1000 °C, exhibit characteristic luminescence (Lum) spectra. These spectra are composed of sharp features attributed to Cr^3+^: ^2^E → ^4^A_2_ transitions (R- and N2-lines at 690 and 696 nm, respectively),^24^ alongside Stokes phonon side bands at 710 and 716 nm overlapping with a broad band at 715 nm corresponding to the Cr^3+^: ^4^T_2_ → ^4^A_2_ transition (Figures 2c and 2d).^25^ This deep red emission can be efficiently excited through ZGO host absorption in the UV (∼260 nm) and via direct Cr^3+^ absorption bands, specifically the ^4^A_2_ → ^4^T1­(^4^P), ^4^A_2_ → ^4^T_1_(^4^F) and ^4^A_2_ → ^4^T_2_(^4^F) transitions located at ∼330, ∼420, and ∼560 nm, respectively ().^26^ No spectral shifts or changes were observed upon mixing of SiO_2_ NPs, which aligns with our XRD findings that indicate no crystalline phase transformation. Interestingly, while the spectral characteristics remain constant, the Lum intensity is significantly affected by the presence of SiO_2_, with a strong dependence on the calcination temperature. For coatings annealed at 800 °C, the Lum intensity systematically decreases with the SiO_2_ content (Figure 2d). This behavior is consistent with a reduction in the effective mass of the active ZGO:Cr material within the layers. Oppositely, coatings calcined 1000 °C first show a notable Lum enhancement, reaching a factor of ∼1.8 for 10% SiO_2_, before decreasing with higher SiO_2_ contents (Figure 2d).

To elucidate the role of the refractive index on the observed Lum changes, we considered that the amount of light emitted by a sample is proportional to both the radiative decay rate (Γ_rad_) and the quantity of emitting material. We calculated Γ_rad_ for the nanocomposite coatings using an expression recently developed for nanoparticle films:^27^

1 $$\Gamma_{\mathrm{rad}}=\Gamma_{0}\times {\left(\frac{n_{\mathrm{eff}}}{n_{\mathrm{np}}}\right)}^{c_{\mathrm{np}}}\times {\left(\frac{3{n_{\mathrm{eff}}}^{2}}{2{n_{\mathrm{eff}}}^{2}+{n_{\mathrm{np}}}^{2}}\right)}^{2}$$

where Γ_0_ is the radiative decay rate in vacuum, *n*_np_ is the refractive index of the emitting nanoparticle (ZGO:Cr), and *c*_np_ is the volume fraction of nanoparticles in the coating. For clarity, the calculated values were normalized to the reference.

As expected, considering the lower refractive index of SiO_2_ relative to ZGO, Γ_rad_ gradually decreases across the series of films calcined at 800 °C. In contrast, for films calcined at 1000 °C, Γ_rad_ increases with the SiO_2_ content up to 30%, where it reaches its maximum value. Consequently, the product of the radiative decay rate and the quantity of ZGO in samples (Γ_rad_·*M*_ZGO_) further clarifies this trend. For samples calcined at 800 °C, Γ_rad_·*M*_ZGO_ decreases with increasing SiO_2_ content. However, for samples calcined at 1000 °C, this product increases by a factor of 1.21 for 10% SiO_2_ before decreasing at higher SiO_2_ concentrations, which remarkably aligns with the observed Lum enhancement values. Furthermore, estimations of the Γ_rad_·*M*_ZGO_ product using PLQY and PL decay measurements strengthens this calculated tendency, demonstrating a 1.41-fold enhancement for 10% SiO_2_. The analysis of time-dependent PL measurements reveals that the total decay rate (Γ_tot_) increases with the SiO_2_ content, as a result of a complex interplay between radiative and nonradiative decay channels (). Additionally, it is worth noting that PL intensity depends not only on Γ_rad_, but also on excitation efficiency and light outcoupling. Consequently, the dependence of Γ_rad_·*M*_ZGO_ on the SiO_2_ content does not exactly match that of PL enhancement; however, it follows the evolution of the refractive index. In summary, by precisely tuning both the Z:S ratio and the calcination temperature, we achieved a fine adjustment of *n*_eff_ in ZGO:Cr PersLNPs-based coatings. This refractive index engineering directly led to significant Lum enhancements without altering the phosphor composition. In the next section, we will demonstrate the effectiveness of our strategy to precisely tailor the afterglow properties of ZGO:Cr PersLNPs-based coatings.

## Afterglow Enhancement

Following excitation, the characteristic deep red PersL from the coatings can be measured. This emission is dominated by the N2 line at 696 nm, which is associated with the Cr^3+^:^2^E → ^4^A_2_ transition in the vicinity of an antisite defect pair (see Figures 3a and 3b).^24,28^ Consistent with our steady-state Lum observations and XRD analysis, the inclusion of SiO_2_ NPs has no discernible influence on the spectral characteristics of the afterglow, but it modifies its intensity. Specifically, for coatings calcined at 800 °C, the integrated PersL intensity shows a monotonous decrease with increasing SiO_2_ content (Figure 2b). Interestingly, for samples calcined at 1000 °C, the integrated PersL exhibits an initial enhancement, reaching a factor of ∼1.7 for 10% SiO_2_, before decreasing at higher SiO_2_ concentrations. This can be explained by the interplay between changes in the Γ_rad_ due to modifications in *n*_eff_ and the amount of active material in the nanocomposite film. At low SiO_2_ content, the partial melting of SiO_2_ increases *n*_eff_, light conversion, and luminescence intensity. However, as the SiO_2_ increases further, the luminescence intensity decreases due to the reduction in the amount of active material in the nanocomposites. This competing behavior is well captured by the product Γ_rad_·*M*_ZGO_, which also peaks at intermediate SiO_2_ content values. Additionally, TL glow curves and PersL decay kinetics show minimal variation across samples, indicating that the trap depth distribution remains largely unaffected by SiO_2_ inclusion (). This trend closely mirrors the behavior observed for the steady-state luminescence intensity, suggesting a common underlying mechanism.

**Figure 3:**
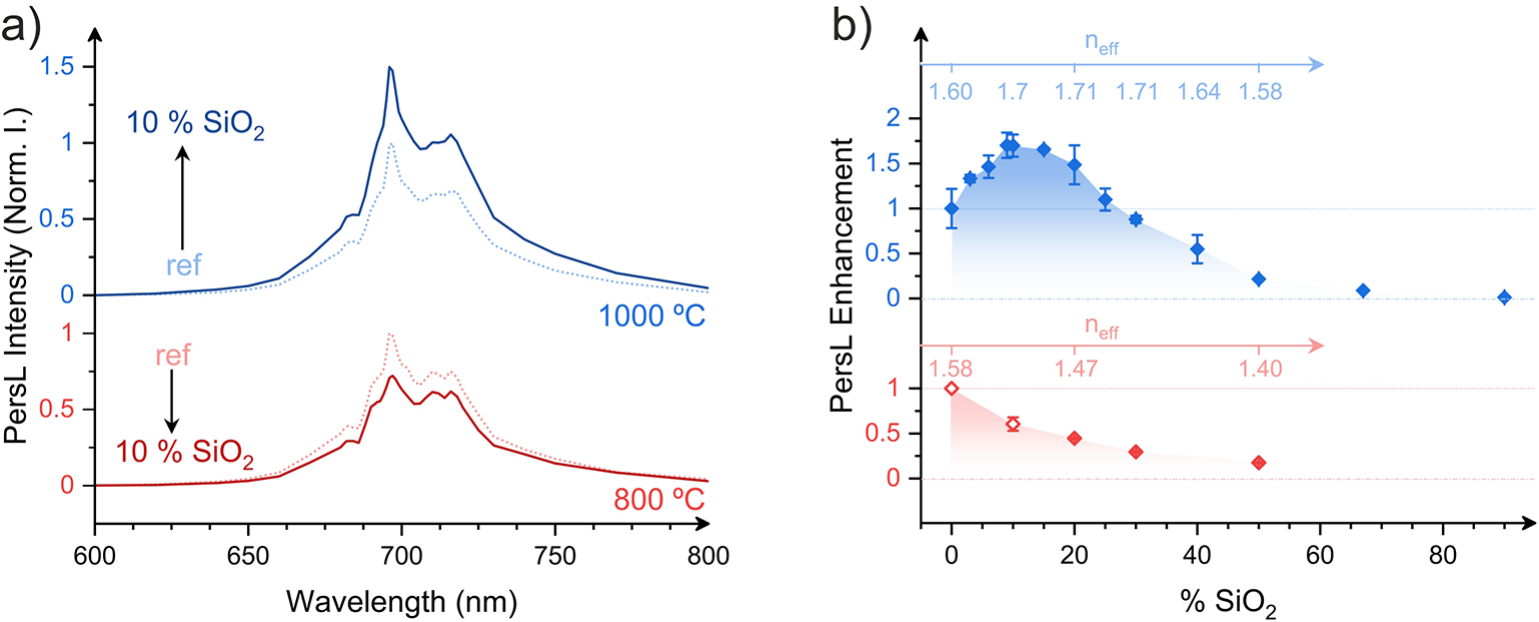
(a) PersL spectra spectra of 100:0 and 90:10 layers calcined at 800 °C (red) and 1000 °C (blue) after UV excitation. (b) PersL enhancement of layers calcined at 800 °C (red) and 1000 °C (blue), represented as a function of the Z:S ratio.

## Charging Rate Modification

The analysis of afterglow charging and release kinetic curves offers a powerful tool to investigate the competing processes that dictate PersL behavior (Figure 4a). The intensity profiles of the nanocomposite films clearly demonstrate that varying the Z:S ratio and the processing temperature not only tunes the afterglow intensity but also profoundly modifies the kinetics of the light emission during excitation (Figures 4b and 4c). This unequivocally highlights the potential of optical environment design to tailor key PersL characteristics without requiring chemical modification of the phosphor itself.

**Figure 4:**
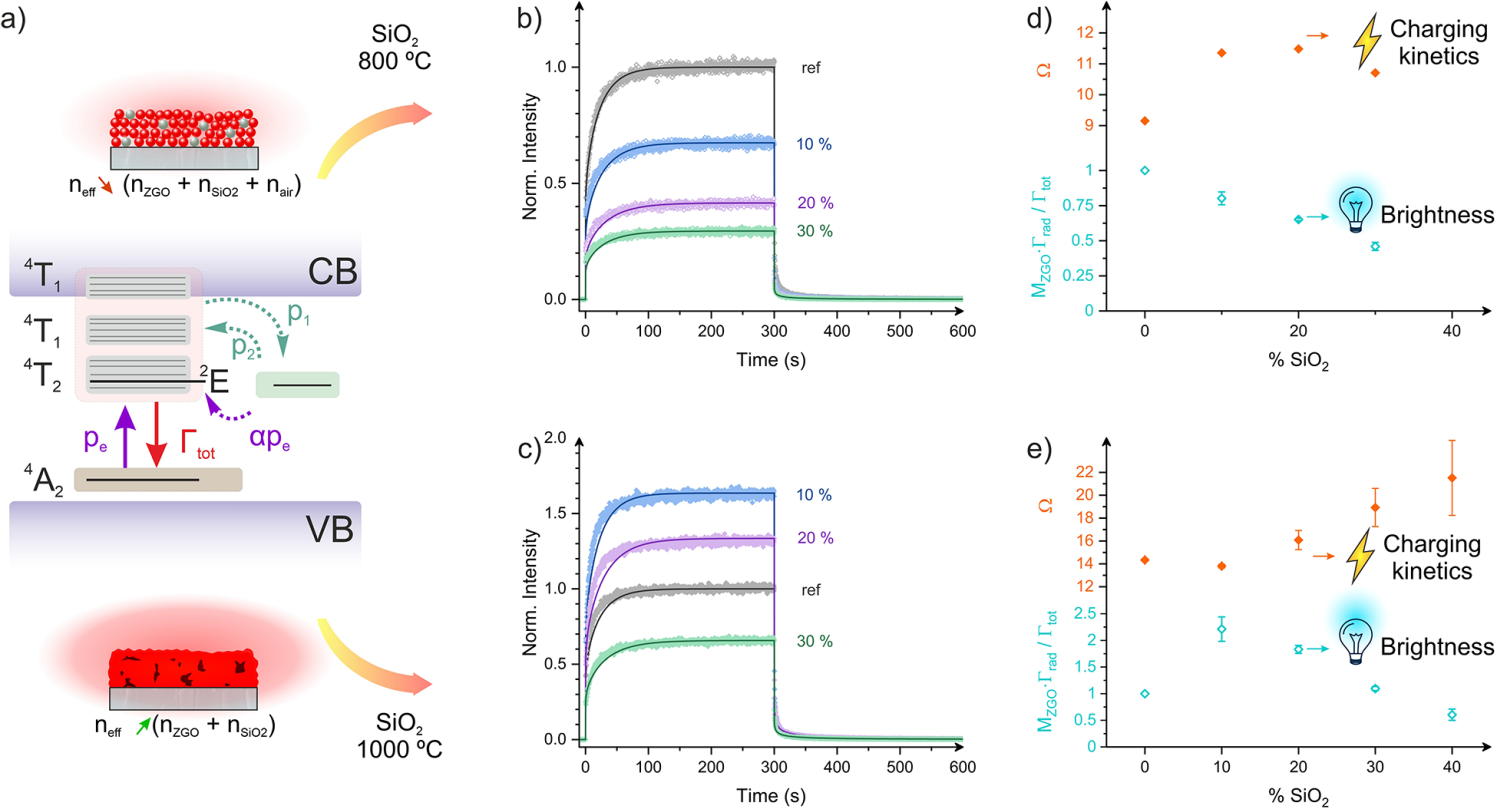
(a) Schematics of the local model used to fit PerL kinetics. Lum and PersL kinetic curves obtained for samples calcined at (b) 800 °C and (c) 1000 °C. Brightness factor and trapping strength parameters used in the model to fit kinetic experimental data represented as a function of SiO_2_ content for samples calcined at (d) 800 and (e) 1000 °C.

To elucidate the origin of the observed tuning of charging and release kinetics, we fitted the Lum and PersL kinetic scans using a recently developed model based on rate equations (see Figures 4b and 4c).^15^ In this model, the total decay rate (Γ_tot_) was fixed based on PL decay curve experiments (). Additionally, very similar release rates (*p*_2_) were estimated from thermoluminescence glow curves measured from the nanocomposite films. Since the inclusion of SiO_2_ had a minimal impact on the thermoluminescence curves, *p*_2_ was assumed to be the same for all samples for the sake of simplicity (). The excitation rate (*p*_e_) and the charging rate (*p*_1_) were set as fitting parameters. Importantly, the proposed model provided excellent fits for all kinetic scans across samples with varying Z:S ratios calcined at both 800 and 1000 °C. The values of the fitting parameters used are listed in . According to these fits, the substantial variation of the scaling factor, which is directly proportional to the Γ_rad_·*M*_ZGO_ product, serves as the primary explanation for the observed kinetic scan intensity variation with the nanocomposite variation and annealing temperature. Indeed, significant scaling factors enhancements (up to a factor of 2.2) were obtained for low SiO_2_ contents in the 1000 °C nanocomposite series, while a consistent decrease was observed across the 800 °C series. These trends show excellent agreement with the Γ_rad_·*M*_ZGO_ product values estimated from the *n*_eff_ modification (see Figures 4d and 4e).

The model also reveals that the optical processes involved in the afterglow (i.e., Γ_tot_ and *p*_e_) are not sufficiently affected by the refractive index modification to fully account for the observed variations in PersL charging and release kinetics. Instead, the fitting results indicate that *p*_1_ is significantly altered across the sample series. Such an alteration in *p*_1_ is attributed to the strain induced by SiO_2_ inclusion in nanocomposite films, a phenomenon akin to effects previously observed under external pressure.^29^ As a direct consequence, the charging strength (Ω), defined as the ratio between *p*_1_ and Γ_tot_, decreases for low SiO_2_ contents in the high-temperature series, whereas it monotonically increases for the low-temperature series. This contributes to explain the observed variations in the afterglow kinetics (Figures 4d and 4e). Notably, the nanocomposite film strategy developed in this study can modulate the intensity and kinetics of PersL without changing the chemical composition of the active afterglow material. This approach holds great promise for engineering smart coatings with customized PersL characteristics.

In this work, we present the first successful demonstration of the refractive index engineering in PersL transparent coatings using a nanocomposite strategy. By incorporating SiO_2_ NPs (with a lower refractive index) into ZGO:Cr PersL suspensions, we fabricated thin, homogeneous coatings approximately 380 nm thick. We show that precisely controlling the nanocomposite composition and calcination temperature enables a broad modulation of the film’s effective refractive index, spanning from 1.40 to 1.71. This wide range is critically achieved by the effective filling of coating voids by molten SiO_2_ at elevated temperatures and directly leads to significant optical enhancement. In excellent agreement with our radiative decay rate calculations, we observed Lum enhancements up to 1.8-fold for coatings containing 10% SiO_2_ calcined at 1000 °C, notably without any alteration to the spectral characteristics. Similarly, the integrated PersL was strongly enhanced (up to 1.7-fold), primarily attributed to the refractive index’s influence on the radiative decay rate. Our kinetic modeling of the charging and release curves revealed that this nanocomposite strategy also uniquely allows for tailoring the afterglow kinetics. This kinetic modulation is a consequence in part of strain induced on the ZGO:Cr nanocrystals by SiO_2_ inclusion, a novel mechanism identified in this context. The ability to precisely tune both afterglow intensity and kinetics without altering the chemical composition of the active PersL material represents a significant advancement. These findings validate our claim that precise tuning of both afterglow intensity and kinetics can be achieved through independent control of strain and refractive index. Experimental measurements and kinetic modeling support this dual-parameter strategy without requiring changes to the phosphor composition. This developed nanocomposite strategy, and more broadly, the application of photonic designs, open bright, i.e., new and exciting avenues for advanced light-emitting and light-storage technologies.

## Methods

### Nanoparticle Fabrication

Spherical ZGO:Cr nanoparticles with a diameter of ∼29 nm were obtained following a microwave-assisted hydrothermal synthesis previously described after adjusting reaction parameters.^22^ Zinc acetate (Sigma–Aldrich, 99.99%, 1.6 mmol), gallium nitrate (Sigma–Aldrich, 99.9%, 3.12 mmol), and chromium nitrate (Sigma–Aldrich, 99%, 7.8 × 10^–2^ mmol) precursors were stirred for 5 min in 104 mL of Milli-Q water. Trisodium citrate (Sigma–Aldrich, 99.5%, 7.8 mmol in 52 mL Milli-Q water) was added to the solution and let stirred at RT for 30 min. Then, tetraetylammonium hydroxide (Sigma–Aldrich) was added dropwise until reaching pH 9 and was allowed to be stirred for another 30 min. The resulting solution was divided in five equal parts that were transferred to five tightly closed Teflon microwave reactors and heated at 220 °C for 30 min using a heating ramp of 10 °C min^–1^. The resulting mixture was washed three times with Milli-Q water and two times with absolute EtOH (VWR Chemicals).

### Film Preparation

To prepare nanocomposites, commercial SiO_2_ nanoparticles (LUDOX) was added in appropriate amount to ZGO:Cr suspension. The resulting nanoparticle mixture was washed two times to obtain suspensions with 30 mg mL^–1^ particle concentration in absolute EtOH. Coatings were obtained by spin coating six portions (150 μL) of as prepared suspension on fused silica substrates with a 1300 rpm speed during 60 s with a 10500 rpm·s^–1^ acceleration ramp. One minute was waited at RT between each deposition. The obtained films were heated on a hot plate at 500 °C for 3 h (10 °C·min^–1^ heat ramp) before being calcined at 800 or 1000 °C for 3 h with a heat ramp of 10 °C min^–1^.

### Structural Characterization

Prior to deposition, nanoparticle size and morphology were inspected using transmission electron microscopy (TEM) (JEOL, Model 2100 Plus), considering hundreds of particles with ImageJ. Coating thickness and homogeneity were studied using a profilometer (Bruker, Model DektakXT Stylus Profilometer, with a 2-μm large stylus and a scanning speed of 200 μm s^–1^) and scanning electron microscopy (SEM) (Hitachi, Model S4800) in the cross-sectional view. The crystalline phase of the coatings was characterized by X-ray diffraction using a Panalytical X’Pert Pro diffractometer (Cu Kα radiation) equipped with a X-Celerator detector in grazing incidence conditions (10° ≤ 2θ ≤ 80°, 0.05° step width, 1 s integration time). The crystalline phase was determined by comparing the obtained XRD diagram with the Powder Diffraction File database. Rietveld refinements of the powder XRD data were carried out using the JANA2006 software to extract precise lattice parameters and calculate the corresponding interplanar spacings.^30^ The peak profiles were modeled using the fundamental parameter approach,^31^ and the background, lattice parameters, atomic positions, atomic displacements, and crystallite size contribution (CsizeG parameter) were refined. The strain associated with each plane was calculated according to ε = (*d* – *d*_0_)/d_0_, where *I* is the refined spacing for the SiO_2_/ZGO nanocomposite and *d*_0_ is the corresponding spacing in the ZGO reference (silica-free) layer. The strain analysis focused on the (404) reflection, which has relatively high intensity and high diffraction angles, providing a reliable signal-to-noise ratio and enhanced sensitivity to small variations in interplanar spacing. By propagating the standard uncertainties of the refined lattice parameter to the calculated interplanar spacings—and, subsequently,—to the strain values uncertainties of ∼1 × 10^–4^ were obtained.

### Optical Characterization

Absorptance spectra were calculated as 1-R-T, using the total transmittance and total reflectance spectra obtained using a UV–vis–NIR spectrophotometer (Cary 7000) coupled to an integrating sphere. The UV–vis–NIR spectrophotometer was also used coupled to a double goniometer (UMA accessory) to measure reflectance and transmittance at three different angles. These spectra were fitted to determine the refractive index of coatings.^27^ Alternatively, the ellipsometry spectra obtained using a commercial variable angle spectroscopic ellipsometer (J.A. Woollam Co., Inc.) were fitted to estimate the refractive index of coatings. A typical accuracy of ±0.02 is estimated for refractive index determination of thin films under standard conditions.

Lum excitation and emission spectra, PersL emission spectra, and kinetic scans, as well as luminescence decay curves, were measured using a commercial spectrophotometer (Edinburgh, Model FLS1000). Before PersL measurements traps were quenched by annealing coatings 150 °C for 5 min and let them cool to RT in dark conditions. PersL kinetic scans were recorded exciting at 330 nm for 5 min. PersL spectra were reconstructed from integrated PersL kinetic scans at different emission wavelengths. A pulsed Xe lamp was used to measure PL decays. Thermoluminescence was measured by a charge-coupled-device (CCD) camera (Roper Pixis 100) coupled with a monochromator (Acton Spectra Pro, Princeton Instruments) on composite powders calcined in the same way as coatings. Since powders do not allow for a fair comparison of absolute TL intensities, we chose to analyze normalized TL glow curves. Temperature was ramped from 15 K to 500 K, using closed-cycle He-flow cryostats (Sumitomo Cryogenics HC-4E) coupled with a heating resistance and a temperature controller (Lakeshore 340).

### Modeling

We fitted the charge/discharge measurements (Figure 4) using analytical solutions derived from a local trapping model that incorporates a trap depth distribution.^15^ Thermoluminescence (TL) measurements were also analyzed using this model, with numerical solutions obtained by assuming linear heating/cooling. All calculations were performed using custom MATLAB codes. Specifically, we used the ode15s function to find numerical solutions and the genetic algorithm and *ga* function to optimize parameters for the best fit to the experimental data.

## Supplementary Material







## Data Availability

The data underlying this study are openly available in the Digital CSIC repository at https://doi.org/10.20350/digitalCSIC/17628. The code used for fitting the ellipsometry and reflectance/transmittance data is provided at https://github.com/Multifunctional-Optical-Materials-Group.

## Notes

Ballistic transmittance and transparency measurements; XRD patterns; ellipsometry spectra and fittings; transmission and reflection measurements; SEM image of SiO_2_ NP thick films; PL excitation spectra, PL decay and PLQY measurements; normalized TL glow curves and time-dependent afterglow decays; results of ellipsometry fits, results of UMA fits, and parameters used to fit luminescence kinetic scans using the local model (PDF)
Transparent Peer Review report available (PDF)
Transparent Peer Review report available (PDF)

## ∇

For V. Castaing: Laboratoire de Physique de la Matière Condensée (PMC), CNRS, Ecole Polytechnique-Institut Polytechnique de Paris, 91120 Palaiseau, France
