# Supplementary material for: Refractive Index and Strain Modulation Tailor the Afterglow of Nanocomposite Films
Source: J Phys Chem Lett. 2025 Oct 23;16(43):11316–23. doi: 10.1021/acs.jpclett.5c02216 (PMC12581153; doi:10.1021/acs.jpclett.5c02216)
Supplement: Supplementary file 1 [file jz5c02216_si_001.pdf]

Refractive index and strain modulation tailor the afterglow of nanocomposite films.

Victor Castaing, Manuel Romero, Théophile Drion, Alberto J. Fernández-Carrión, Gabriel Lozano* and Hernán Míguez.*

Institute of Materials Science of Seville, Spanish National Research Council – University of Seville, C/ Américo Vespucio 49, 41092 Seville, Spain.

*Correspondence should be addressed to: VC (victor.castaing@icmse.csic.es) or GL (g.lozano@csic.es)

Supporting Information.

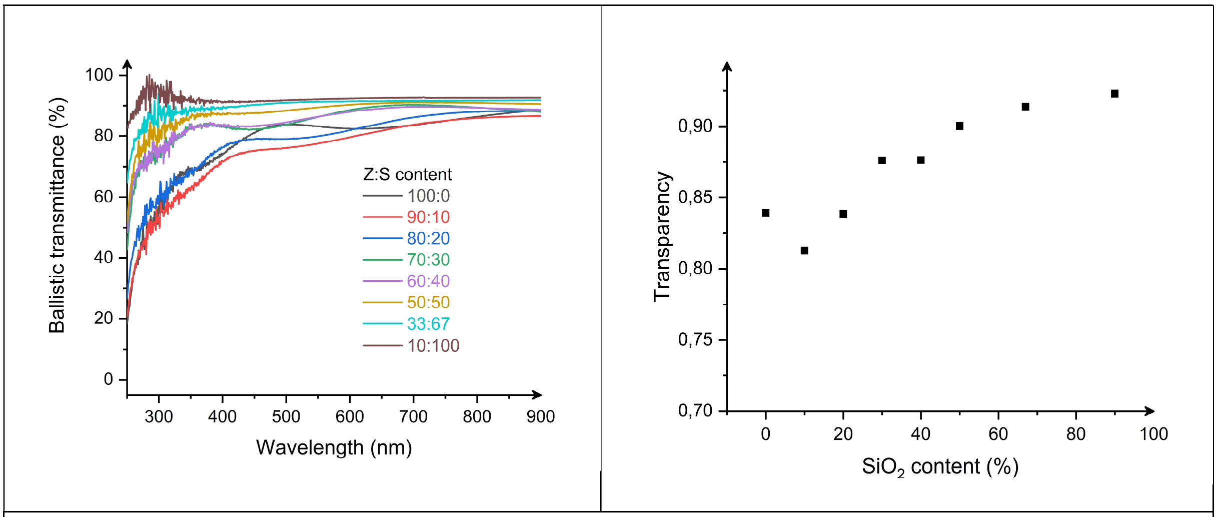

Figure S1: Ballistic transmittance (T_b) of the composite films annealed at 1000 °C (left). Transparency of the films obtained from $\int_{400}^{900} T_b(\lambda)d\lambda$ as a function of SiO_2 content.

The changes observed in the transmittance spectra originate from two main effects. First, the inclusion of SiO_2 results in denser films with an effective refractive index closer to that of the fused silica substrate. This reduces specular reflectance and scattering, thereby increasing transmittance, particularly in the visible range. Second, ZGO:Cr exhibits optical absorption due

to Cr^{3+} transitions and ZGO host absorption, which are more pronounced below 500 nm. Consequently, nanocomposite films with a smaller proportion of ZGO nanoparticles are anticipated to exhibit greater transmission in the UV region.

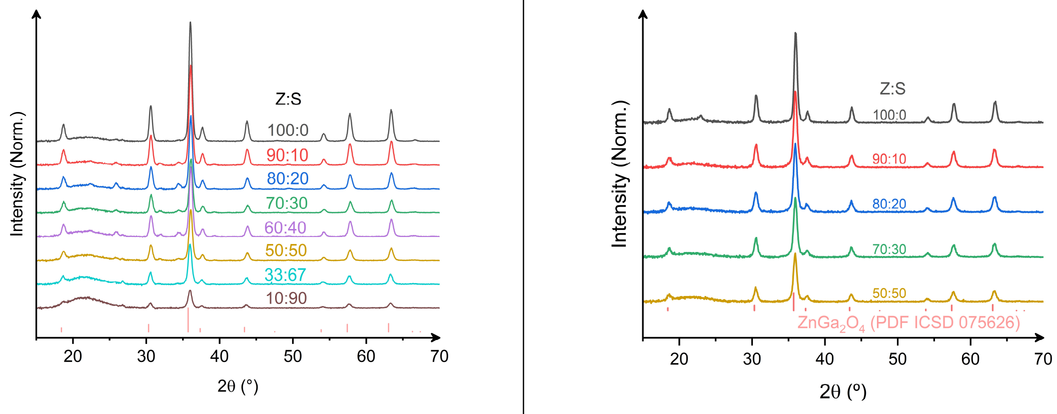

Figure S2: XRD diagram of composite films annealed at 1000 °C (left) and 800 °C (right).

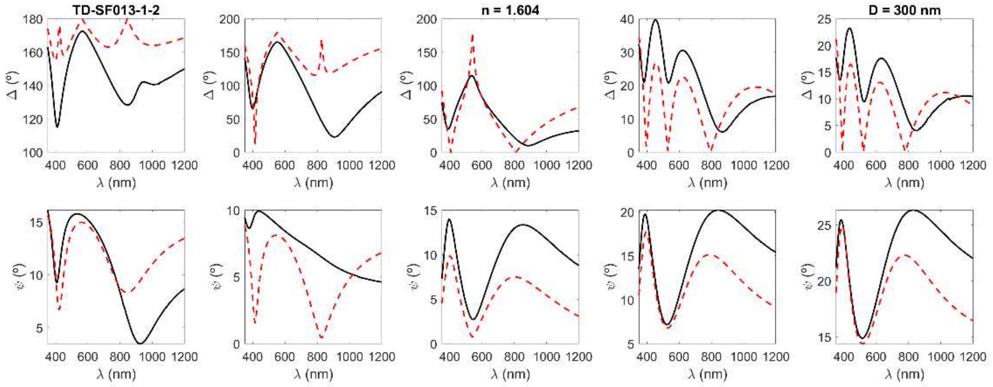

Figure S3a: Ellipsometry spectra (plain, black) and fittings (dashed, red) of ZGO:Cr film annealed at 1000 °C.

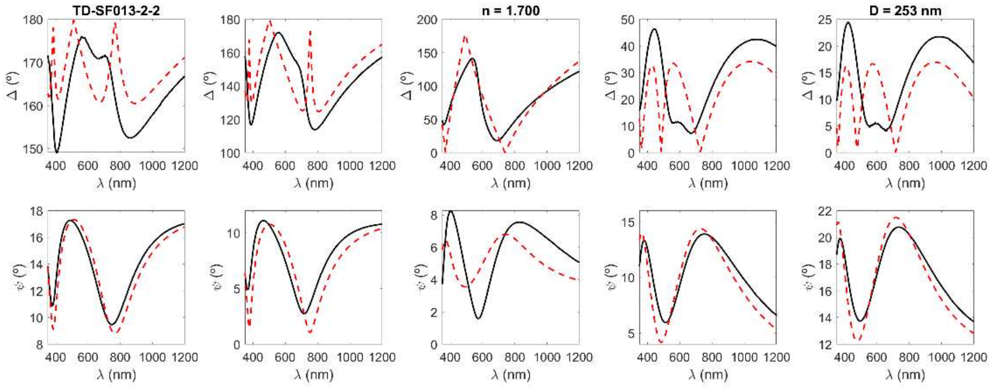

Figure S3b: Ellipsometry spectra (plain, black) and fittings (dashed, red) of 90:10 composite film annealed at 1000 °C.

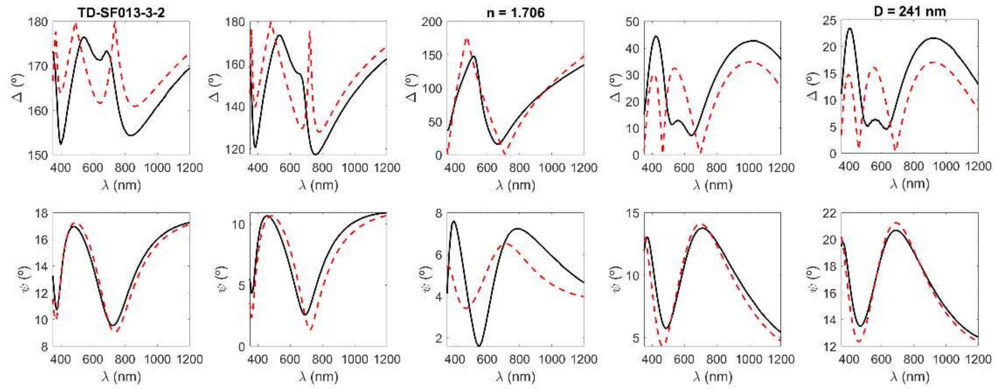

Figure S3c: Ellipsometry spectra (plain, black) and fittings (dashed, red) of 80:20 composite film annealed at 1000 °C.

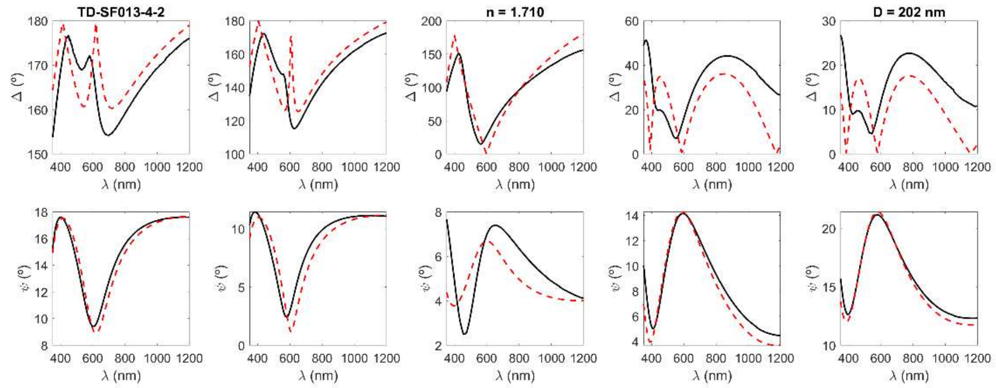

Figure S3d: Ellipsometry spectra (plain, black) and fittings (dashed, red) of 70:30 composite film annealed at 1000 °C.

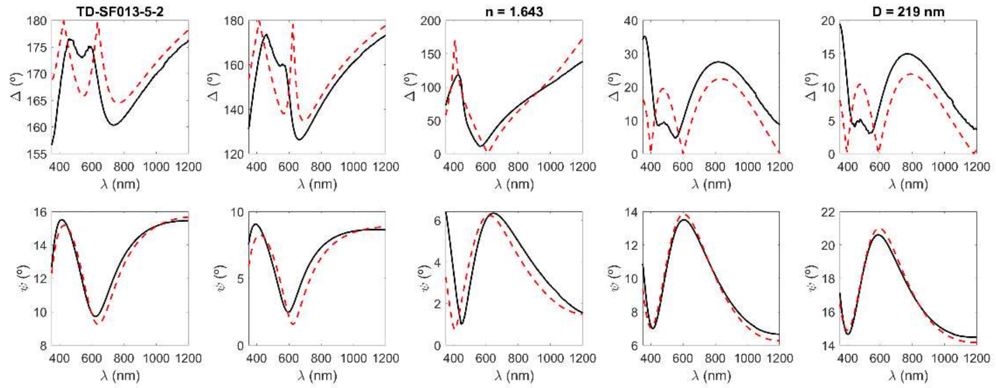

Figure S3e: Ellipsometry spectra (plain, black) and fittings (dashed, red) of 60:40 composite film annealed at 1000 °C.

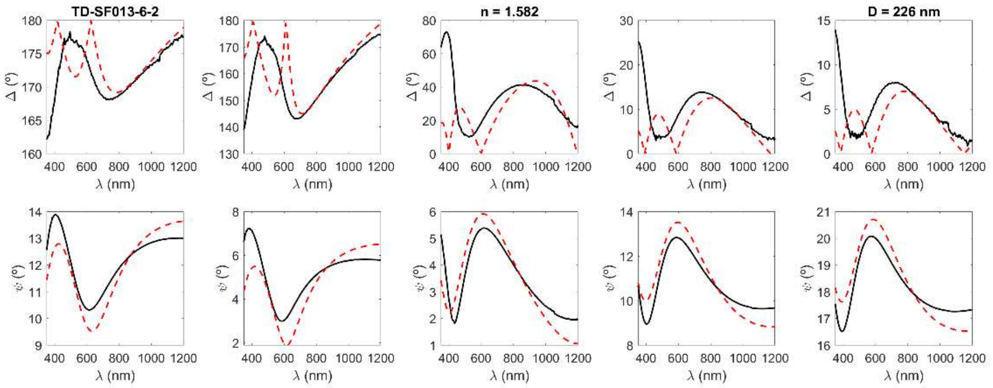

Figure S3f: Ellipsometry spectra (plain, black) and fittings (dashed, red) of 50:50 composite film annealed at 1000 °C.

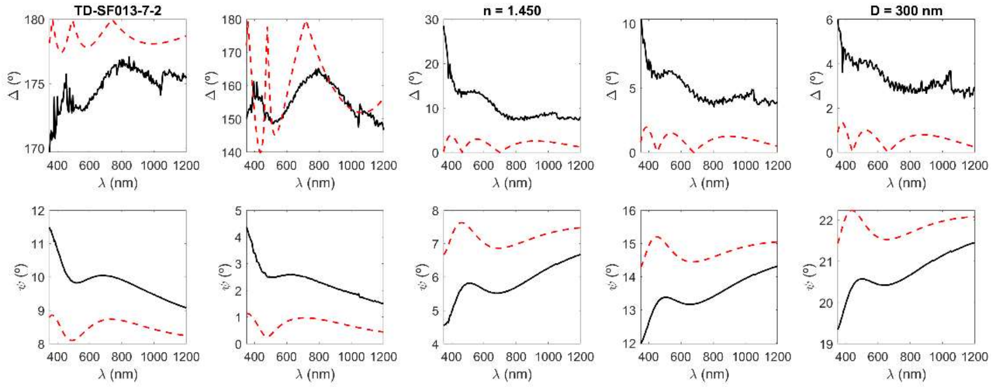

Figure S3g: Ellipsometry spectra (plain, black) and fittings (dashed, red) of 33:67 composite film annealed at 1000 °C.

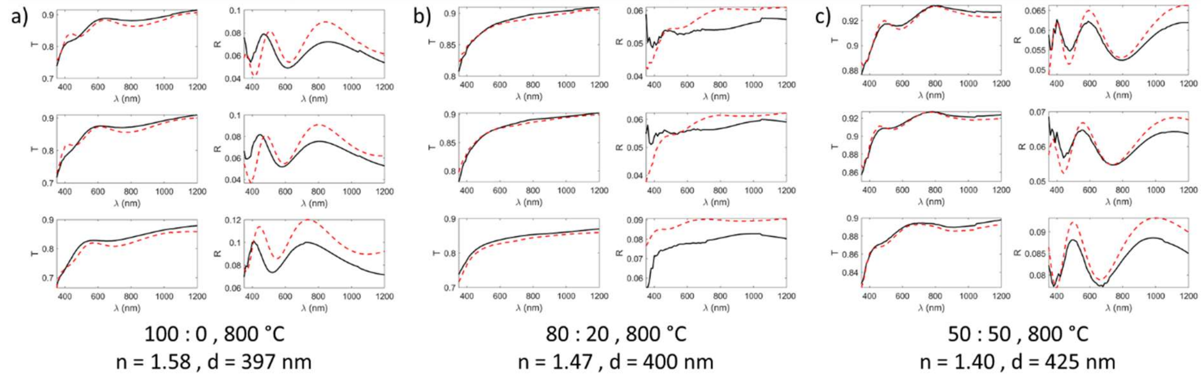

Figure S4: Transmission and reflection spectra (plain, black) obtained using the UMA and fittings (dashed, red) of composite films annealed at 800 °C.

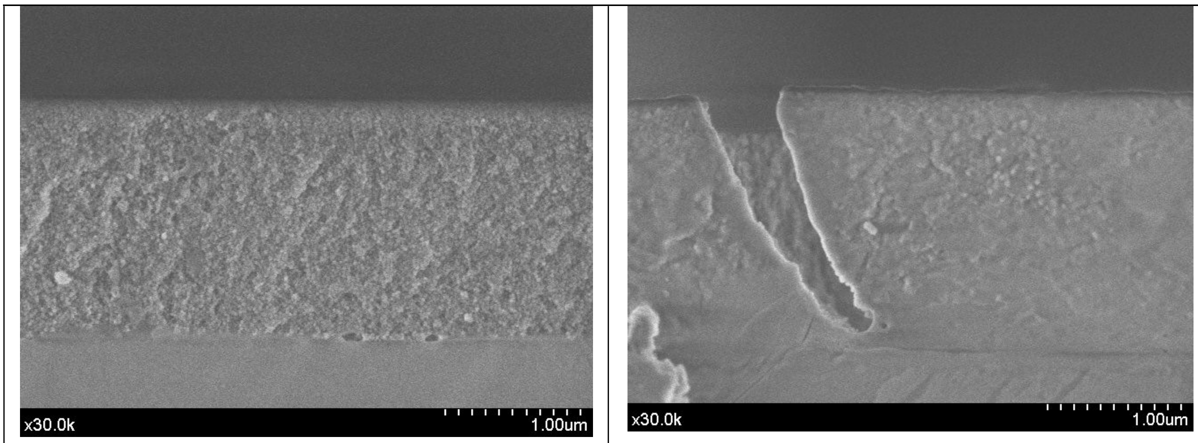

Figure S5: SiO_2 NPs thick films calcined at 800 °C (left) and 1000 °C (right).

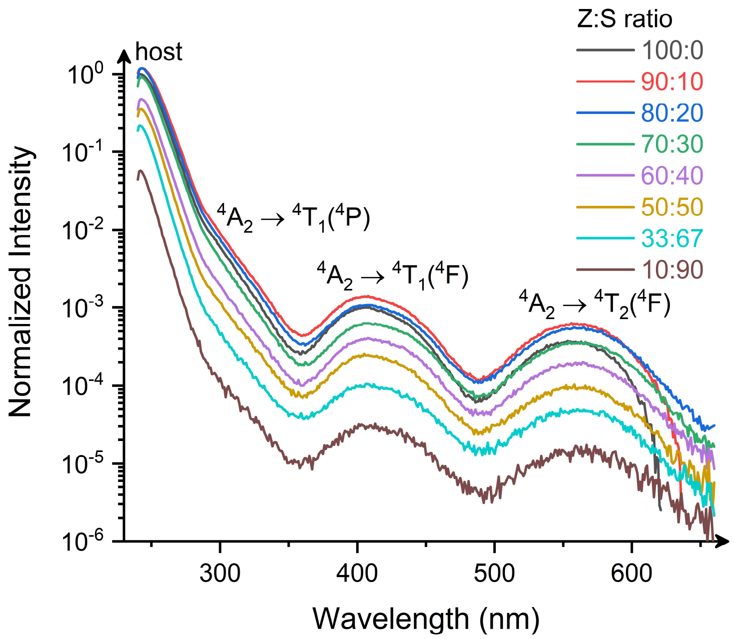

Figure S6: Excitation spectra of ZGO:Cr (Cr^{3+}: $^2E \rightarrow {}^4A_2$ emission) of composite films annealed at 1000 °C.

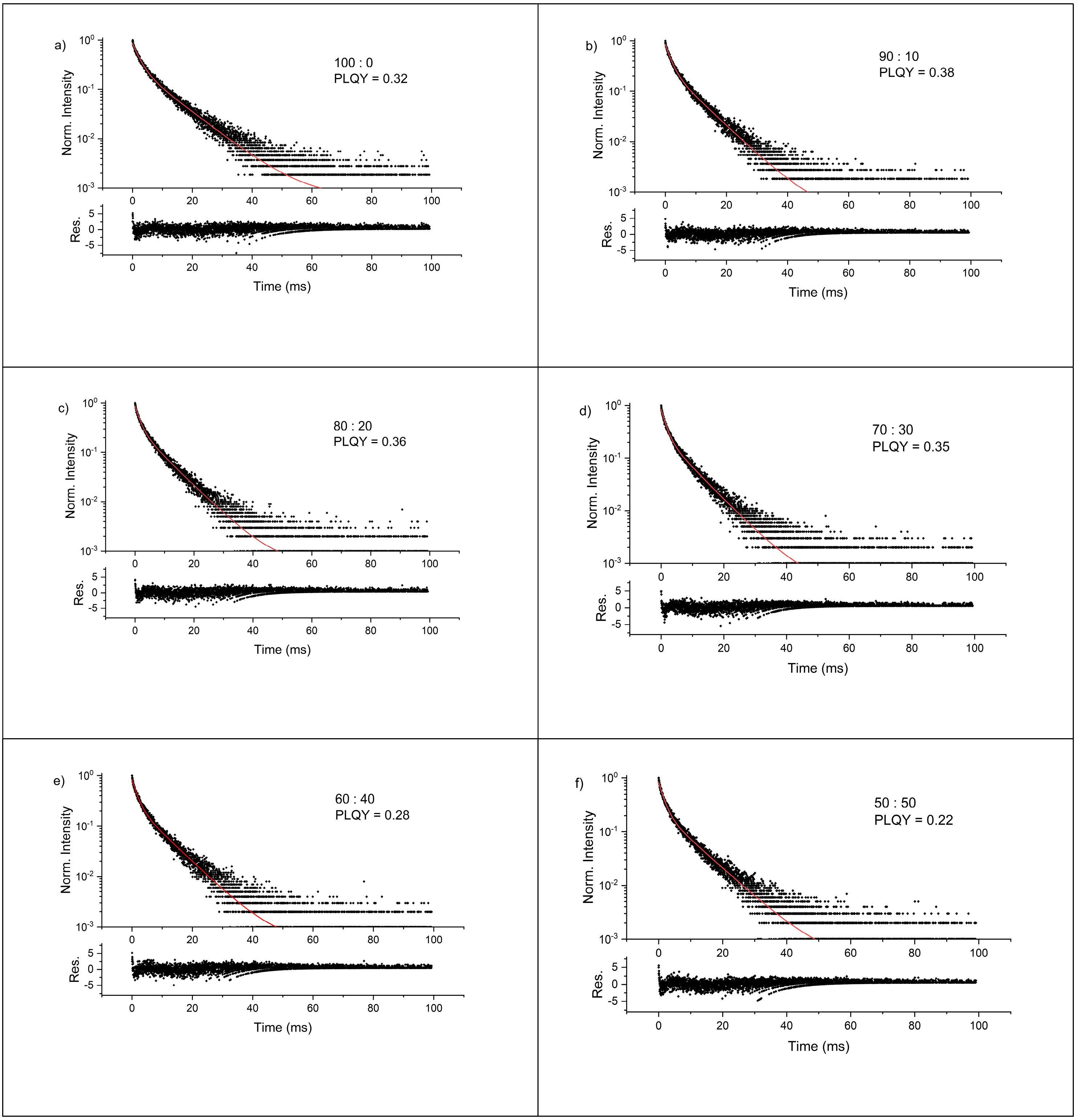

a)
100 : 0
PLQY = 0.32
b)
90 : 10
PLQY = 0.38
c)
80 : 20
PLQY = 0.36
d)
70 : 30
PLQY = 0.35
e)
60 : 40
PLQY = 0.28
f)
50 : 50
PLQY = 0.22
Norm. Intensity
Res.
Time (ms)

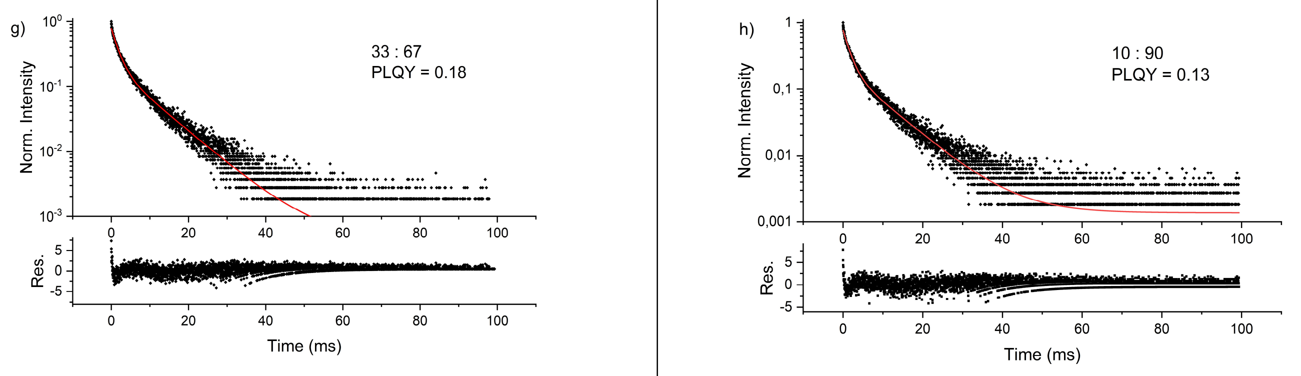

Figure S7: Photoluminescence decay of a) 100 : 0, b) 90 : 10, c) 80 : 20, d) 70 : 30, e) 60 : 40, f) 50 : 50, g) 33 : 67 and h) 10 : 90 composite films. PLQY values are given in inset.

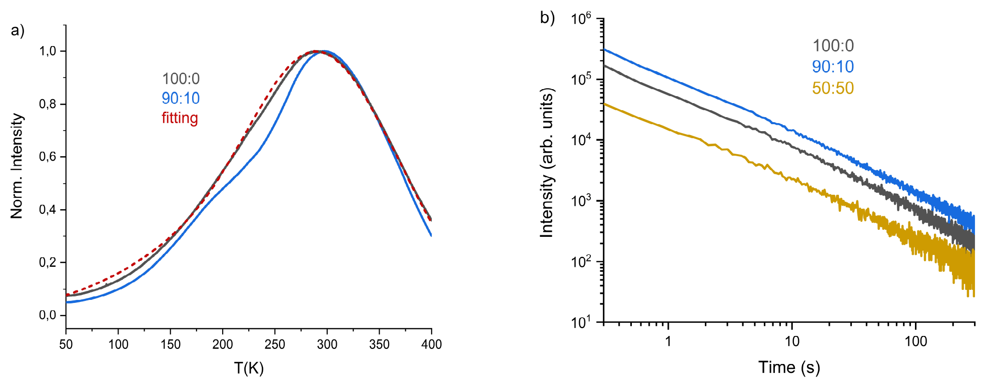

Figure S8: a) Normalized TL glow curves of 100:0 and 90:10 composite films annealed at 1000 °C. The dashed red line corresponds to the fitting used to reproduce the time-dependent luminescence at room temperature. b) Time-dependent afterglow intensity for nanocomposites with different amounts of SiO_2.

Table S1: Results of ellipsometry fits of films calcined at 1000 °C.

% SiO_2 nominal	n_{eff}	Thickness (nm)	ZGO	SiO_2	Air	Average Δ RMSE	Average ψ RMSE
0	1.60	300	68 %	0 %	32 %	24.5	3.93
10	1.70	253	65 %	27 %	8 %	12.4	1.28
20	1.71	241	63 %	30 %	7 %	11.5	0.963
30	1.71	202	60 %	38 %	2 %	11.2	0.855

40	1.64	219	48 %	48 %	4 %	9.37	0.579
50	1.58	226	30 %	70 %	0 %	8.35	0.671
67	1.45	300	1 %	99 %	0 %	5.71	1.39

Table S2: Results of UMA fits of films calcined at 800 °C.					
% SiO_2 nominal	n_{eff}	Thickness (nm)	ZGO	SiO_2	Air
0	1.58	398	66 %	0 %	34 %
20	1.47	400	40 %	30 %	30 %
50	1.40	425	30 %	35 %	35 %

Table S3: Parameters used to fit Lum and PersL kinetic scans using the local model.						
Composite	$T_{calc.}$ (°C)	Γ_{tot} (Hz)	p_e (Hz)	p_1 (Hz)	Ω	Scaling factor
100:0	1000	215	0.601 ± 0.064	3081 ± 26	14.3 ± 0.1	1
90:10	1000	267	0.600 ± 0.034	3691 ± 34	13.8 ± 0.2	2.212 ± 0.229
80:20	1000	268	0.514 ± 0.056	4315 ± 34	16.1 ± 0.8	1.831 ± 0.075
70:30	1000	302	0.512 ± 0.054	5724 ± 505	18.9 ± 1.7	1.096 ± 0.038
60:40	1000	280	0.512 ± 0.054	6015 ± 917	21.5 ± 3.3	0.604 ± 0.106
100:0	800	206	0.368 ± 0.043	1889 ± 73	9.2 ± 0.4	1
90:10	800	279	0.321 ± 0.002	3165 ± 588	11.4 ± 2.1	0.802 ± 0.046
80:20	800	292	0.321 ± 0.002	3360 ± 706	11.5 ± 2.4	0.652 ± 0.008
70:30	800	313	0.333 ± 0.019	3267 ± 541	10.7 ± 2.0	0.460 ± 0.029
