# Supplementary material for: Refractive Index and Strain Modulation Tailor the Afterglow of Nanocomposite Films
Source: J Phys Chem Lett. 2025 Oct 23;16(43):11316–23. doi: 10.1021/acs.jpclett.5c02216 (PMC12581153; doi:10.1021/acs.jpclett.5c02216)
Supplement: Supplementary file 3 [file jz5c02216_si_003.pdf]

jz-2025-022163.R1

Name: Peer Review Information for "Refractive index and strain modulation tailor the afterglow of nanocomposite films"

First Round of Reviewer Comments

Reviewer: 1

Comments to the Author

The manuscript presents a study on the synthesis of Cr^{3+} doped $ZnGa_2O_4$ nanoparticle by a microwave-assisted hydrothermal synthesis. The authors claimed that the incorporation of SiO_2 effectively modulates both the refractive index from 1.45 to 1.7 and strain effects in the nanocomposite thin films, resulting in a 1.7-fold enhancement in luminescence intensity and persistent luminescence performance. The data was well interpreted and presented. However, some details need further clarity, as questioned in below.

In summary, I think that the paper is publishable in The Journal of Physical Chemistry Letters after a minor revision.

Questions:

1. Page 5, line 19, the author claimed that the film thickness reduction after 1000°C heat treatment is attributed to the melting of SiO_2. However, the possibility of thickness reduction due to volatilization of SiO_2 or nanoparticles must be ruled out. Therefore, the TGA curve of the composite film should be provided.

2. Page 6, line 7, the XRD refinement analysis reveals that the introduction of SiO_2 can induces strain of $ZnGa_2O_4$ nanoparticles. How the strain was quantified?

3. Page 8, line 16, quantitative analysis shows the film porosity significantly decreased between 0% and 8% after 1000°C heat treatment. However, the specific testing procedures and raw data should be provided. What kind of measurement was conducted?

4. Page 9, line 15, the author claimed that the luminescent intensity of the 10% SiO_2 incorporated sample increased by 1.7-fold after 1000°C heat treatment. However, the PL QY only improved by 6% (Figure S7 a, b). Therefore, this discrepancy should be explained.

How much was the PL intensity at low temperature (liquid N_2) when compared with the room-temperature one?

Reviewer: 2

Comments to the Author

The authors present tailoring the afterglow of nanocomposite films by modulating refractive index and stain of films with the help of SiO_2 loading. However, the mechanism of observed results is not convincing. The fitting of ellipsometry data also needs to be rechecked for better fitting curves and values. The authors should address the following concerns more carefully to achieve the merits of this journal.

1) The after glow is mainly affected with the trapping characteristics and local perturbation due to structural or compositional changes eventually affect trap characterstics and therefore more detailed experimental results and analysis are required for defect analysis. The TL/OSL results should be presented in more details for better understanding of mechanism.

2) How the SiO_2 affect the characterstics Cr^{3+} decay times? The mechanism need to be explained.

3) Effect of strain and refractive index are separate or dependent parameters to affect the properties.

4) How the ballistic transmission was measured and why the transmission below 400 nm is mainly affected by SiO_2 ratio and it is close to 90% transmission after 500 nm. The transmission curve is not well explained and not correlated with other results.

5) The refractive index calculation fitting is the major concern as the variation of refractive index is the major output of the article while the fitting of ellipsometry data is not at par. Goodness of fitting (rms, reduced chi2 values etc.) should be also presented all the fitting.

6) It is also necessary to give the error bars on the values as the articles conclusion relies on the effect of these values.

7) Why closely matching SiO_2 nanoparticles are necessary to prepare the films?

8) It is also not understood why the films were sintered at two temperatures. It is obvious that more transparency will be obtained at higher temperature than why lower temperature sintering was investigated. The advantage of sintering at 800 degC is not clear.

9) The error bars on strain values also necessary.

10) The relation between porosity and refractive index should be also presented. This relation may also help to understand the variation of refractive index which is maximum for a particular ratio.

11) The increment in luminescence and product of decay rate also need to be explained. Why one value increase by a factor of 1.8 and another by 1.21. How these values aligns trend wise. The mechanism should be explained.

12) Trend of values (maximum at particular value while another decrease with SiO2 ratio) is also need to be explained with a better mechanism.

13) The role of trap centres is the most important factor for persistent luminescence and therefore should be presented for all samples with detailed analysis. In place of having results for 800 deg C, it may be more useful to have defects related analysis in details.

14) Whether the TL intensity was normalised to the PL intensity as samples having higher luminescence efficiency will give higher intensity of TL glow peaks as well.

15) Persistent luminescence should be presented, in terms of remaining intensity after particular time for all samples. The initial Persistent luminescence intensity is not as important as its variation with time. Initial intensity may be high but doesn't have practical applications if decays faster.

16) The claim of "Precisely tuning of afterglow and kinetics" is not supported with the available data and mechanism.

Author's Response to Peer Review Comments:

Response le er to reviewers

Reviewer #1 (R1)

R1: The manuscript presents a study on the synthesis of Cr^{3+} doped $ZnGa_2O_4$ nanopar cle by a microwave-assisted hydrothermal synthesis. The authors claimed that the incorpora on of SiO_2 effec vely modulates both the refrac ve index from 1.45 to 1.7 and strain effects in the nanocomposite thin films, resul ng in a 1.7-fold enhancement in luminescence

intensity and persistent luminescence performance. The data was well interpreted and presented. However, some details need further clarity, as ques oned in below. In summary, I think that the paper is publishable in The Journal of Physical Chemistry Le ers a er a minor revision.

Authors (A): We are pleased that the reviewer supports the publica on of our results. We appreciate their comments, which are addressed below.

R1: 1) Page 5, line 19, the author claimed that the film thickness reduc on a er 1000°C heat treatment is a ributed to the mel ng of SiO_2. However, the possibility of thickness reduc on due to vola liza on of SiO_2 or nanopar cles must be ruled out. Therefore, the TGA curve of the composite film should be provided.

A: We understand the concern of the reviewer but it is technically challenging to perform TGA measurements on the nanocomposite films as the rela ve of silica nanopar cles respect to the total weight of the substrate plus the film is very low. To rule out the possibility of sublima on, we conducted a control experiment in which a thick film composed solely of SiO_2 nanopar cles was annealed at 1000 °C under the same condi ons used for the nanocomposites. Scanning electron microscopy SEM images (see Figure R1) clearly reveal the presence of a con nuous SiO_2 layer a er annealing, indica ng that the silica nanopar cles do not evaporate under these condi ons. The presence of Na in the composi on of commercial SiO_2 nanopar cles (LUDOX), in turn, can act as a fundent at high temperatures. This explains the densifica on of the composite film and the associated reduc on in thickness. We have addressed this point in the revised manuscript in response to the reviewer's concern (see page 4, line79).

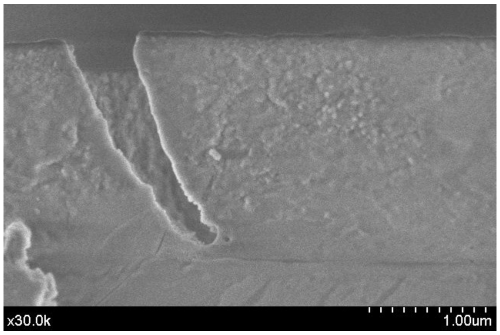

Figure R1. SEM image of a cross sec on of a SiO_2 nanopar cle film annealed at 1000 ºC.

R1: 2) Page 6, line 7, the XRD refinement analysis reveals that the introduc on of SiO_2 can induces strain of $ZnGa_2O_4$ nanopar cles. How the strain was quan fied?

A: Rietveld refinements of the powder XRD data were performed using the JANA2006 so ware to extract precise la ce parameters and calculate the corresponding interplanar spacings. The peak profiles were modeled using the fundamental parameter approach [R. W. Cheary and A. Coelho, J. Appl. Crystallogr., 1992, 25, 109–121], and the background, la

ce parameters, atomic posi ons, atomic displacements, and crystallite size contribu on (CsizeG parameter) were refined. The strain associated with each plane was calculated according to $\varepsilon = (d - d_0)/d_0$, where d is the refined spacing for the $SiO_2/ZnGa_2O_4$ nanocomposite and d_0 is the corresponding spacing in the $ZnGa_2O_4$ (ZGO) reference (silica-free) layer. The strain analysis focused on the (404) reflec on, which has rela vely high intensity and high diffrac on angles. This provides a reliable signal-to-noise ra o and enhanced sensi vity to small varia ons in interplanar spacing. We clarified this in the new version of the manuscript, following the reviewer's recommenda on (see page 5, line 87 and Methods).

R1: 3) Page 8, line 16, quan ta ve analysis shows the film porosity significantly decreased between 0% and 8% a er 1000°C heat treatment. However, the specific tes ng procedures and raw data should be provided. What kind of measurement was conducted?
A: The porosity values reported in the manuscript were es mated from the effec ve refrac ve index (n_{eff}) obtained through ellipsometry fi ngs. To extract the filling frac on (ff) of $ZnGa_2O_4$, SiO_2, and air, we applied the Bruggeman effec ve medium approxima on, using the known bulk refrac ve indices of $ZnGa_2O_4$ (n = 1.94), SiO_2 (n = 1.46), and air (n = 1.00). This approach allowed us to quan fy the rela ve ff of each component in the nanocomposite films. The detailed fi ng results, including the es mated porosity values, are provided in Table S1 of the Suppor ng Informa on. We have further clarified the approach we followed in the new version of the manuscript (see page 7, line 118). Finally, we would like to emphasize that if the manuscript is accepted, the raw measurement data will be made available through DIGITAL.CSIC, an open online ins tu onal repository. This is explained in the Data Statement sec on of the manuscript.

R1: 4) Page 9, line 15, the author claimed that the luminescent intensity of the 10% SiO2 incorporated sample increased by 1.7-fold a er 1000°C heat treatment. However, the PLQY only improved by 6% (Figure S7 a, b). Therefore, this discrepancy should be explained. How much was the PL intensity at low temperature (liquid N2) when compared with the room-temperature one? A: We agree that the apparent discrepancy between the improvement in PLQY and the enhancement in luminescence intensity requires clarifica on. Luminescence intensity depends on three factors: the material's ability to absorb excita on light, its ability to convert absorbed photons into emi ed photons (i.e., PLQY), and its ability to outcouple the light generated within the film. In the case highlighted by the reviewer, the 10% SiO_2 nanocomposite sample annealed at 1000 °C exhibited reduced porosity and increased densifica on. These changes could lead to more efficient excita on and emission outcoupling. These effects explain the 1.7-fold increase in luminescence intensity observed despite the modest change in PLQY. We did not perform temperature-dependent PL measurements in this study. However, we agree that such an analysis could provide

valuable insights and will consider including it in future work. We have addressed this interes ng point in the amended version of the manuscript (see page 9, line 159).

Reviewer #2 (R2)

R2: The authors present tailoring the a erglow of nanocomposite films by modula ng refrac ve index and stain of films with the help of SiO2 loading. However, the mechanism of observed results is not convincing. The fi ng of ellipsometry data also needs to be rechecked for be er fi ng curves and values. The authors should address the following concerns more carefully to achieve the merits of this journal.
Authors (A): We are confident that we can address the reviewer's concerns and make our results suitable for publica on in JPCL.

R2: 1) The a erglow is mainly affected with the trapping characteris cs and local perturba on due to structural or composi onal changes eventually affect trap characteris cs and therefore more detailed experimental results and analysis are required for defect analysis. The TL/OSL results should be presented in more details for be er understanding of mechanism.
A: Our primary focus was modula ng the intensity of persistent luminescence (PersL) through refrac ve index engineering. We do not expect this process to affect the intrinsic trap characteris cs of ZGO:Cr. However, we acknowledge that thermoluminescence (TL) measurements are central to any study of PersL materials. Indeed, the TL glow curves of the nanocomposite films show similar profiles, regardless of the inclusion of SiO_2, sugges ng that the trap depth distribu on remains largely intact. Consequently, the a erglow decay dynamics remain nearly iden cal across all samples (see Fig. R2a). Based on this observa on, we modeled all nanocomposites using the same trap depth distribu on (see Fig. R2b) and successfully fit both the TL and PersL kine cs of the different samples studied simultaneously. Following the reviewer's recommenda on, we have included a fi ng of a TL curve (see Figure S8a). We have also expanded the related discussion in the new version of the revised manuscript (see page 11, line 184 and page 12, line 201).

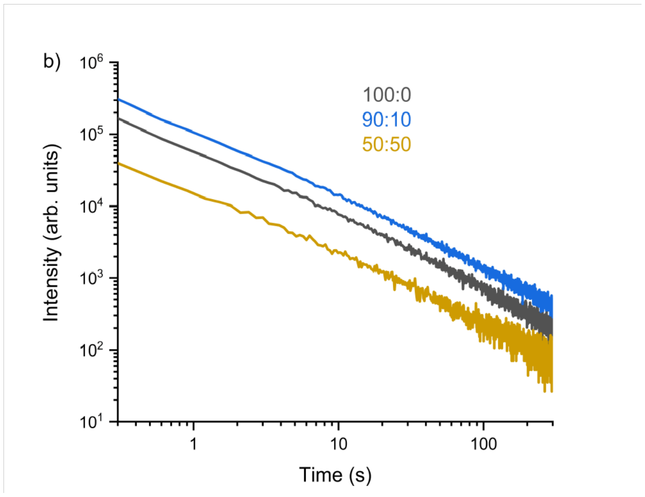

Figure R2a. Time-dependent a erglow intensity for nanocomposites with different amounts of SiO_2.

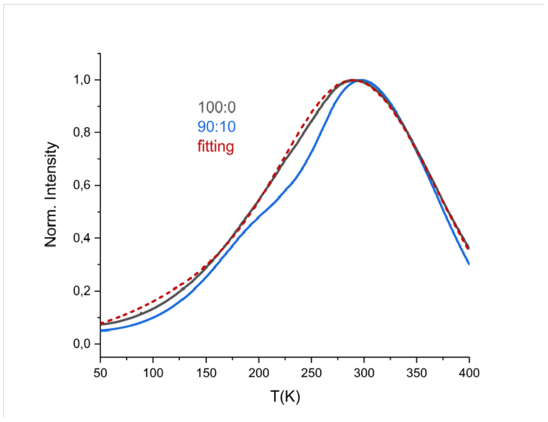

Figure R2b Normalized thermoluminescence glow curve for the 0% (black solid line) and 10% (blue solid line) SiO_2 sample annealed at 1000ºC. The red dashed line corresponds to the fi ng used to reproduce the me-dependent luminescence at room temperature.

R2: 2) How the SiO_2 affect the characters cs Cr3+ decay mes? The mechanism needs to be explained.

A: Table R1 shows the decay rates extracted from the me-dependent luminescence measurements presented in Figure S7. The decay rate generally increases with SiO_2 content; however, the underlying mechanisms differ depending on the annealing temperature. The increase in decay rate observed for samples annealed at 1000 °C is primarily due to an increase in the radia ve decay rate. This enhancement correlates with an increase in the effec ve refrac ve index caused by film densifica on. Similarly, samples annealed at 800 °C are expected to decrease their radia ve decay rate as the effec ve refrac ve index decreases with SiO_2. However, the inclusion of SiO_2 introduces addi onal non-radia ve pathways, likely due to interfacial effects or local disorder, counterac ng the effect on the radia ve rate. These nonradia ve decay channels outweigh the expected reduc on in the

radia ve rate associated with the lower refrac ve index of nanocomposites processed at 800 °C. We have clarified this point further in the revised manuscript, following the reviewer's advice (see page 9, line 159).

Composition ($ZGO:SiO_2$)	$T_{calc.}$ (°C)	Γ_{tot} (Hz)
100:0	1000	215
90:10	1000	267
80:20	1000	268
70:30	1000	302
60:40	1000	280
100:0	800	206
90:10	800	279
80:20	800	292
70:30	800	313

Table R1: Experimental decay rates for samples annealed at 1000ºC and 800ºC with different amounts of SiO_2.

R2: 3) Effect of strain and refrac ve index are separate or dependent parameters to affect the proper es.

A: Although the inclusion of SiO_2 alters the effec ve refrac ve index (n_{eff}) and strain, the mechanism by which it modifies the proper es of PersL has a different physical origin. Strain is produced by mechanical forces resul ng from the mismatch in thermal expansion coefficients between ZGO and SiO_2 nanopar cles during annealing. This strain can affect the local crystal field and poten ally modify the radia ve decay rate, as well as the trapping and detrapping processes that govern PersL. On the other hand, changing the refrac ve index of the nanocomposite cons tuents (n_{SiO2}, n_{ZGO}) or their respec ve loading frac on (ff_{SiO2}, ff_{ZGO}) tunes the n_{eff} of the nanocomposite film, thus affec ng the local density of states (LDOS) and, consequently, the radia ve rate of the emi er. In summary, changes in the refrac ve index are expected to primarily influence the radia ve decay rate, while strain may impact the kine cs of charge trapping and release. We have further clarified this point in the revised version of the manuscript (see page 14, line 242).

R2: 4) How the ballis c transmission was measured and why the transmission below 400 nm is mainly affected by SiO_2 ra o and it is close to 90% transmission a er 500 nm. The transmission curve is not well explained and not correlated with other results.

A: Ballis c transmission measurements were carried out using a Cary 5000 (Agilent) spectrometer coupled with a double goniometer (UMA accessory). This setup allows for angleresolved transmi ance and reflectance measurements. The changes observed in the transmi ance spectra originate from two main effects. First, the inclusion of SiO_2 results in denser films with an effec ve refrac ve index closer to that of the fused silica substrate. This reduces specular reflectance and sca ering, thereby increasing transmi ance, par cularly in the visible range. Second, ZGO:Cr exhibits op cal absorp on due to Cr^{3+} transi ons and ZGO host absorp on, which are more pronounced below 500 nm. Consequently, nanocomposite films with a smaller propor on of ZGO nanopar cles are an cipated to exhibit greater transmission in the UV region. We have further discussed this point in the amended version of the manuscript (see the discussion below Figure S1).

R2: 5) The refrac ve index calcula on fi ng is the major concern as the varia on of refrac ve index is the major output of the ar cle while the fi ng of ellipsometry data is not at par. Goodness of fi ng (rms, reduced chi2 values etc.) should be also presented all the fi ng. A: We understand the reviewer's concern about the accuracy of determining the refrac ve index, a central aspect of our work. In fact, calcula ng the effec ve refrac ve index in our system is par cularly challenging due to the nature of the samples: transparent thin films with refrac ve indices close to those of the fused silica substrate. This similarity reduces the contrast in the ellipsometry signal, making the fi ng process more sensi ve to experimental noise. Nevertheless, the ellipsometry fi ngs are accurate enough to confirm the refrac ve index trend across the sample series. To improve reliability, we performed each fit simultaneously for five different incident angles and two polariza ons. This helps constrain the model and reduce ambiguity. While individual measurements may not perfectly match the fit, the global trend across all angles is fairly well reproduced. To further support our findings, we have included root mean square error (RMSE) values for each fit in the new version of the Suppor ng Informa on (see Table R2 and Table S1). These metrics quan ta vely assess the quality of the fits and confirm that the extracted refrac ve index values are robust.

Composite	$T_{calc.}$ (°C)	Average Δ RMSE	Average ψ RMSE
100:0	1000	24.5	3.93
90:10	1000	12.4	1.28
80:20	1000	11.5	0.963
70:30	1000	11.2	0.855

60:40	1000	9.37	0.579
50 :50	1000	8.35	0.671
33 :67	1000	5.71	1.39

Table R2: RMSE values of the ellipsometry fi ngs.

R2: 6) It is also necessary to give the error bars on the values as the ar cle conclusion relies on the effect of these values.

A: We agree that error es mates are important for evalua ng the reliability of experimental data. However, the parameters obtained from refrac ve index and composi on values derived from spectroscopic fi ngs are obtained through numerical op miza on rather than direct measurements. Therefore, standard error bars cannot be provided. Nevertheless, we can es mate a typical accuracy of ±0.02 for refrac ve index determina on of thin films under standard condi ons. We have addressed this issue in the revised version of the manuscript (see Methods).

R2: 7) Why closely matching SiO_2 nanopar cles are necessary to prepare the films?

A: Although it is not strictly necessary, using size-matched (monodisperse) nanopar cles when preparing nanocomposite films helps ensure homogeneity and transparency. Mixing nanopar cles of significantly different sizes can lead to agglomera on during film forma on, resul ng in inhomogeneous coa ngs of poor op cal quality. For this reason, we selected ZGO and SiO_2 nanopar cles with similar diameters to promote uniform dispersion and minimize sca ering. We have commented on this point in the amended manuscript (see page 3, line 59).

R2: 8) It is also not understood why the films were sintered at two temperatures. It is obvious that more transparency will be obtained at higher temperature than why lower temperature sintering was inves gated. The advantage of sintering at 800 ºC is not clear.

A: Our goal was not to maximize transparency; rather, we sought to explore how thermal treatment condi ons influence the effec ve refrac ve index of the films, a factor central to our strategy for tuning luminescence proper es. Annealing at 1000 °C par ally melts the SiO_2 nanopar cles, enabling them to fill the voids between the ZGO nanopar cles. This densifica on increases the effec ve refrac ve index at low SiO_2 content and decreases it at higher content, enabling a broad, non-monotonic tuning range. In contrast, annealing at 800 °C leaves the SiO_2 nanopar cles in the nanocomposite films intact. Consequently, films with high SiO_2 content retain their original porosity, and the effec ve refrac ve index decreases smoothly as high-index ZGO nanopar cles are replaced by low-index SiO_2. This lower-temperature treatment allows us to achieve refrac ve index values that cannot be reached at 1000 °C, par cularly in the low-index range. Combining both annealing condi ons allows

for wider, more controlled modula on of the refrac ve index, which is essen al for the tuning of the PersL proper es demonstrated in this work. We have further clarified this in the revised manuscript (see page 7, line 122).

R2: 9) The error bars on strain values also necessary.
A: Error bars were obtained by propaga ng the standard uncertain es of the refined la ce parameter to the calculated interplanar spacings and, subsequently, to the strain values, leading to uncertain es of ~1×10^{-4}. Since the calculated strain values are on the order of 10^{-2} (e.g., 0.0198), the associated errors are at least two orders of magnitude smaller and therefore not visible in the graphs, as they fall within the symbol size. We have commented on this in the new version of the manuscript (see Methods).

R2: 10) The rela on between porosity and refrac ve index should be also presented. This rela on may also help to understand the varia on of refrac ve index which is maximum for a par cular ra o.
A: Understanding the rela onship between porosity and refrac ve index is central to interpre ng the trends observed in our nanocomposite films. In our study, we extracted the effec ve refrac ve index by fi ng spectroscopic measurements using a Bruggeman effec ve medium approxima on. This model enabled us to es mate the filling frac on (ff) of ZGO, SiO_2, and air (porosity) in the films. At 1000 °C, the par al mel ng of SiO_2 nanopar cles leads to the densifica on of the films, which reduces porosity and increases the refrac ve index for low SiO_2 content. As the SiO_2 content increases, the propor on of low-index material dominates and the refrac ve index begins to decrease. At 800 °C, the shape of the SiO_2 nanopar cles is preserved, and the porosity remains rela vely high. This results in a smoother, monotonic decrease in the refrac ve index as the SiO_2 content increases, reaching values lower than those a ainable at 1000 °C. The interplay between densifica on and composi on explains the non-monotonic refrac ve index behavior observed at 1000 °C and the monotonic trend at 800 °C. We have clarified this rela onship and the use of the Bruggeman model in the revised manuscript (see page 7, line 108 and page 7, line 118).

R2: 11) The increment in luminescence and product of decay rate also need to be explained. Why one value increase by a factor of 1.8 and another by 1.21. How these values align trend wise. The mechanism should be explained.
A: Enhancements in luminescence intensity and radia ve decay rate, corrected for the amount of ac ve material (M_{ZGO}), follow similar trends. However, they are not expected to match exactly, as emission intensity is also affected by film morphology and light sca ering. These factors may influence light incoupling and outcoupling. As the referee points out, the 10% SiO_2 sample annealed at 1000 °C exhibits a 1.8-fold increase in luminescence, while the calculated increase in the product of the radia ve decay rate and the amount of ac ve

material (Γ_{rad}·M_{ZGO}) is ~1.21fold. Addi onally, PLQY and decay measurements (see Figure S7 in the Suppor ng Informa on) show a 1.41-fold enhancement for the same sample, which lies between the calculated and observed values. We a ribute this discrepancy to the difference in the sca ering proper es of the nanocomposite compared to the reference ZGO film. As previously men oned in this response le er, sca ering affects the incoupling and outcoupling of light. This can lead to more effec ve excita on and improved light extrac on. We have addressed this issue in the revised manuscript (see page 9, line 159).

R2: 12) Trend of values (maximum at par cular value while another decrease with SiO_2 ra o) is also need to be explained with a be er mechanism.
A: As previously discussed, the luminescence and persistent luminescence intensi es exhibit non-monotonic behavior with respect to the SiO_2 content, especially for samples annealed at 1000 °C. This trend is primarily governed by the interplay between changes in the radia ve rate due to modifica ons in the effec ve refrac ve index and the amount of ac ve material in the film composi on. At low SiO_2 content, the par al mel ng of SiO_2 nanopar cles results in denser films and an increased effec ve refrac ve index. This enhances the radia ve decay rate, improving light genera on and luminescence intensity. However, as the SiO_2 increases further, the luminescence intensity decreases due to the reduc on in the amount of ac ve material in the nanocomposite films. This compe ng behavior is well captured by the product Γ_{rad}·M_{ZGO}, which also peaks at intermediate SiO_2 content values. We have clarified this point further in the new version of the manuscript, following the reviewer's advice (see page 11, line 179).

R2: 13) The role of trap centers is the most important factor for persistent luminescence and therefore should be presented for all samples with detailed analysis. In place of having results for 800 ºC, it may be more useful to have defects related analysis in details.
A: We agree that the trap landscape plays a central role in determining a erglow behavior. In our study, we examined this phenomenon using TL measurements. As the TL data show (see Fig. S8 of the Suppor ng Informa on), the inclusion of SiO_2 has a minimal impact on the TL glow curves. This suggests that the distribu on of trap depths in ZGO remains largely unaffected by the presence of SiO_2 nanopar cles in the nanocomposite films. Consequently, the persistent luminescence decay kine cs remain nearly unchanged (see Fig. R2a). We have commented on this in the amended version of the manuscript (see page 12, line 201).

R2: 14) Whether the TL intensity was normalised to the PL intensity as samples having higher luminescence efficiency will give higher intensity of TL glow peaks as well.
A: We agree with the reviewer that samples with stronger a erglow also have stronger TL signals because both phenomena are governed by trap popula on dynamics. However, the

limita ons of the experimental setup we used for this part of the characteriza on do not allow for a fair comparison of absolute TL intensi es. For this reason, we chose to analyze TL glow curves that were normalized in intensity. We have clarified this point in the revised manuscript (see page 17, line 307).

R2: 15) Persistent luminescence should be presented, in terms of remaining intensity a er par cular me for all samples. The ini al Persistent luminescence intensity is not as important as its varia on with me. Ini al intensity may be high but doesn't have prac cal applica ons if decays faster.

A: In our study, we focused on analyzing the integrated PersL signal during the a erglow period. As the me-dependent PersL intensity measurements displayed in Fig. R2a (new Figure S8b), all samples exhibit very similar a erglow decay profiles regardless of the SiO_2 content or processing condi ons. This indicates that the trap distribu on and release dynamics remain largely unchanged across the different nanocomposite films, as discussed in our response le er. Consequently, the a erglow intensity enhancement factor remains nearly constant throughout the a erglow period. We have further clarified this point in the revised manuscript (see page 11, line 184 and new Figure S8b).

R2: 16) The claim of "Precisely tuning of a erglow and kine cs" is not supported with the available data and mechanism.

A: We respec ully disagree with the reviewer. We are convinced that the data and analysis presented in the manuscript support the claim of precise tuning of a erglow intensity and kine cs. The tuning of a erglow intensity is clearly demonstrated by the systema c varia on of the effec ve refrac ve index. This is supported by experimental measurements and theore cal modeling. Regarding the kine cs, we used a validated rate equa on model to fit the luminescence and persistent luminescence charging curves. The results of these fits revealed that the charging rate (p_1) varies with SiO_2 content and annealing temperature while the trap release rate (p_2) remains constant. This suggests that strain induced by SiO_2 inclusion affects charging dynamics without altering trap depth distribu on. Based on these findings, we demonstrate that our nanocomposite strategy enables independent modula on of the intensity and kine cs of persistent luminescence without altering the ZGO composi on, represen ng a landmark in the field.
