# Supplementary material for: Refractive Index and Strain Modulation Tailor the Afterglow of Nanocomposite Films
Source: J Phys Chem Lett. 2025 Oct 23;16(43):11316–23. doi: 10.1021/acs.jpclett.5c02216 (PMC12581153; doi:10.1021/acs.jpclett.5c02216)
Supplement: Supplementary file 2 [file jz5c02216_si_002.pdf]

Response letter to reviewers

Reviewer #1 (R1)

R1: The manuscript presents a study on the synthesis of Cr^{3+} doped $ZnGa_2O_4$ nanoparticle by a microwave-assisted hydrothermal synthesis. The authors claimed that the incorporation of SiO_2 effectively modulates both the refractive index from 1.45 to 1.7 and strain effects in the nanocomposite thin films, resulting in a 1.7-fold enhancement in luminescence intensity and persistent luminescence performance. The data was well interpreted and presented. However, some details need further clarity, as questioned in below. In summary, I think that the paper is publishable in The Journal of Physical Chemistry Letters after a minor revision.

Authors (A): We are pleased that the reviewer supports the publication of our results. We appreciate their comments, which are addressed below.

R1: 1) Page 5, line 19, the author claimed that the film thickness reduction after 1000°C heat treatment is attributed to the melting of SiO_2. However, the possibility of thickness reduction due to volatilization of SiO_2 or nanoparticles must be ruled out. Therefore, the TGA curve of the composite film should be provided.

A: We understand the concern of the reviewer but it is technically challenging to perform TGA measurements on the nanocomposite films as the relative of silica nanoparticles respect to the total weight of the substrate plus the film is very low. To rule out the possibility of sublimation, we conducted a control experiment in which a thick film composed solely of SiO_2 nanoparticles was annealed at 1000 °C under the same conditions used for the nanocomposites. Scanning electron microscopy SEM images (see Figure R1) clearly reveal the presence of a continuous SiO_2 layer after annealing, indicating that the silica nanoparticles do not evaporate under these conditions. The presence of Na in the composition of commercial SiO_2 nanoparticles (LUDOX), in turn, can act as a fundent at high temperatures. This explains the densification of the composite film and the associated reduction in thickness. We have addressed this point in the revised manuscript in response to the reviewer's concern (see page 4, line79).

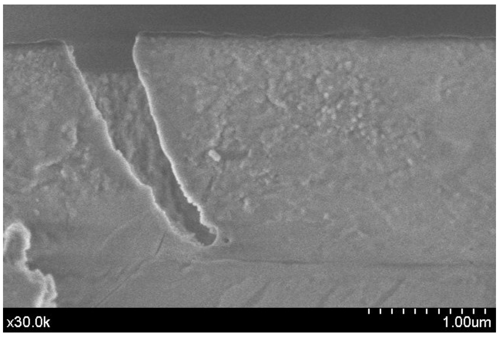

Figure R1. SEM image of a cross section of a SiO_2 nanoparticle film annealed at 1000 ºC.

R1: 2) Page 6, line 7, the XRD refinement analysis reveals that the introduction of SiO_2 can induces strain of $ZnGa_2O_4$ nanoparticles. How the strain was quantified?

A: Rietveld refinements of the powder XRD data were performed using the JANA2006 software to extract precise lattice parameters and calculate the corresponding interplanar spacings. The peak profiles were modeled using the fundamental parameter approach [R. W. Cheary and A. Coelho, *J. Appl. Crystallogr.*, 1992, 25, 109–121], and the background, lattice parameters, atomic positions, atomic displacements, and crystallite size contribution (CsizeG parameter) were refined. The strain associated with each plane was calculated according to $\varepsilon = (d - d_0)/d_0$, where d is the refined spacing for the $SiO_2/ZnGa_2O_4$ nanocomposite and d_0 is the corresponding spacing

in the $ZnGa_2O_4$ (ZGO) reference (silica-free) layer. The strain analysis focused on the (404) reflection, which has relatively high intensity and high diffraction angles. This provides a reliable signal-to-noise ratio and enhanced sensitivity to small variations in interplanar spacing. We clarified this in the new version of the manuscript, following the reviewer's recommendation (see page 5, line 87 and Methods).

R1: 3) Page 8, line 16, quantitative analysis shows the film porosity significantly decreased between 0% and 8% after 1000°C heat treatment. However, the specific testing procedures and raw data should be provided. What kind of measurement was conducted?

A: The porosity values reported in the manuscript were estimated from the effective refractive index (n_{eff}) obtained through ellipsometry fittings. To extract the filling fraction (ff) of $ZnGa_2O_4$, SiO_2, and air, we applied the Bruggeman effective medium approximation, using the known bulk refractive indices of $ZnGa_2O_4$ (n = 1.94), SiO_2 (n = 1.46), and air (n = 1.00). This approach allowed us to quantify the relative ff of each component in the nanocomposite films. The detailed fitting results, including the estimated porosity values, are provided in Table S1 of the Supporting Information. We have further clarified the approach we followed in the new version of the manuscript (see page 7, line 118). Finally, we would like to emphasize that if the manuscript is accepted, the raw measurement data will be made available through DIGITAL.CSIC, an open online institutional repository. This is explained in the Data Statement section of the manuscript.

R1: 4) Page 9, line 15, the author claimed that the luminescent intensity of the 10% SiO2 incorporated sample increased by 1.7-fold after 1000°C heat treatment. However, the PLQY only improved by 6% (Figure S7 a, b). Therefore, this discrepancy should be explained. How much was the PL intensity at low temperature (liquid N2) when compared with the room-temperature one?

A: We agree that the apparent discrepancy between the improvement in PLQY and the enhancement in luminescence intensity requires clarification. Luminescence intensity depends on three factors: the material's ability to absorb excitation light, its ability to convert absorbed photons into emitted photons (i.e., PLQY), and its ability to outcouple the light generated within the film. In the case highlighted by the reviewer, the 10% SiO_2 nanocomposite sample annealed at 1000 °C exhibited reduced porosity and increased densification. These changes could lead to more efficient excitation and emission outcoupling. These effects explain the 1.7-fold increase in luminescence intensity observed despite the modest change in PLQY. We did not perform temperature-dependent PL measurements in this study. However, we agree that such an analysis could provide valuable insights and will consider including it in future work. We have addressed this interesting point in the amended version of the manuscript (see page 9, line 159).

Reviewer #2 (R2)

R2: The authors present tailoring the afterglow of nanocomposite films by modulating refractive index and stain of films with the help of SiO2 loading. However, the mechanism of observed results is not convincing. The fitting of ellipsometry data also needs to be rechecked for better fitting curves and values. The authors should address the following concerns more carefully to achieve the merits of this journal.

Authors (A): We are confident that we can address the reviewer's concerns and make our results suitable for publication in JPCL.

R2: 1) The afterglow is mainly affected with the trapping characteristics and local perturbation due to structural or compositional changes eventually affect trap characteristics and therefore more detailed experimental results and analysis are required for defect analysis. The TL/OSL results should be presented in more details for better understanding of mechanism.

A: Our primary focus was modulating the intensity of persistent luminescence (PersL) through refractive index engineering. We do not expect this process to affect the intrinsic trap characteristics of ZGO:Cr. However, we acknowledge that thermoluminescence (TL) measurements are central to any study of PersL materials. Indeed, the TL glow curves of the nanocomposite films show similar profiles, regardless of the inclusion of SiO_2, suggesting that the trap depth distribution remains largely intact. Consequently, the afterglow decay dynamics remain nearly identical across all samples (see Fig. R2a). Based on this observation, we modeled all nanocomposites using the same trap depth distribution (see Fig. R2b) and successfully fit both the TL and PersL kinetics of the different samples studied simultaneously. Following the reviewer's recommendation, we have included a fitting of a TL curve (see Figure S8a). We have also expanded the related discussion in the new version of the revised manuscript (see page 11, line 184 and page 12, line 201).

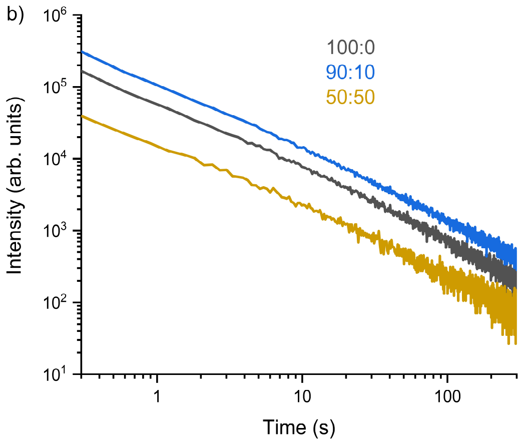

Figure R2a. Time-dependent afterglow intensity for nanocomposites with different amounts of SiO_2.

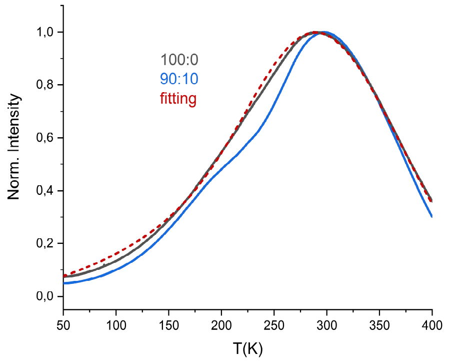

Figure R2b Normalized thermoluminescence glow curve for the 0% (black solid line) and 10% (blue solid line) SiO_2 sample annealed at 1000ºC. The red dashed line corresponds to the fitting used to reproduce the time-dependent luminescence at room temperature.

R2: 2) How the SiO_2 affect the characterstics Cr3+ decay times? The mechanism needs to be explained.

A: Table R1 shows the decay rates extracted from the time-dependent luminescence measurements presented in Figure S7. The decay rate generally increases with SiO_2 content; however, the underlying mechanisms differ depending on the annealing temperature. The increase in decay rate observed for samples annealed at 1000 °C is primarily due to an increase in the radiative decay rate. This enhancement correlates with an increase in the effective refractive index caused by film densification. Similarly, samples annealed at 800 °C are expected to decrease their radiative decay rate as the effective refractive index decreases with SiO_2. However, the inclusion of SiO_2 introduces additional non-radiative pathways, likely due to interfacial effects or local disorder, counteracting the effect on the radiative rate. These non-radiative decay channels outweigh the expected reduction in the radiative rate associated with the lower refractive index of nanocomposites processed at 800 °C. We have clarified this point further in the revised manuscript, following the reviewer's advice (see page 9, line 159).

Composition ($ZGO:SiO_2$)	$T_{calc.}$ (°C)	Γ_{tot} (Hz)
100:0	1000	215
90:10	1000	267
80:20	1000	268
70:30	1000	302
60:40	1000	280
100:0	800	206
90:10	800	279
80:20	800	292
70:30	800	313

Table R1: Experimental decay rates for samples annealed at 1000ºC and 800ºC with different amounts of SiO_2.

R2: 3) Effect of strain and refractive index are separate or dependent parameters to affect the properties.

A: Although the inclusion of SiO_2 alters the effective refractive index (n_{eff}) and strain, the mechanism by which it modifies the properties of PersL has a different physical origin. Strain is produced by mechanical forces resulting from the mismatch in thermal expansion coefficients between ZGO and SiO_2 nanoparticles during annealing. This strain can affect the local crystal field and potentially modify the radiative decay rate, as well as the trapping and detrapping processes that govern PersL. On the other hand, changing the refractive index of the nanocomposite constituents (n_{SiO2}, n_{ZGO}) or their respective loading fraction (ff_{SiO2}, ff_{ZGO}) tunes the n_{eff} of the nanocomposite film, thus affecting the local density of states (LDOS) and, consequently, the radiative rate of the emitter. In summary, changes in the refractive index are expected to primarily influence the radiative decay rate, while strain may impact the kinetics of charge trapping and release. We have further clarified this point in the revised version of the manuscript (see page 14, line 242).

R2: 4) How the ballistic transmission was measured and why the transmission below 400 nm is mainly affected by SiO_2 ratio and it is close to 90% transmission after 500 nm. The transmission curve is not well explained and not correlated with other results.

A: Ballistic transmission measurements were carried out using a Cary 5000 (Agilent) spectrometer coupled with a double goniometer (UMA accessory). This setup allows for angle-resolved transmittance and reflectance measurements. The changes observed in the transmittance spectra originate from two main effects. First, the inclusion of SiO_2 results in denser films with an effective refractive index closer to that of the fused silica substrate. This reduces specular reflectance and scattering, thereby increasing transmittance, particularly in the visible range. Second, ZGO:Cr exhibits optical absorption due to Cr^{3+} transitions and ZGO host absorption, which are more pronounced below 500 nm. Consequently, nanocomposite films with a smaller proportion of ZGO nanoparticles are anticipated to exhibit greater transmission in the UV region. We have further discussed this point in the amended version of the manuscript (see the discussion below Figure S1).

R2: 5) The refractive index calculation fitting is the major concern as the variation of refractive index is the major output of the article while the fitting of ellipsometry data is not at par. Goodness of fitting (rms, reduced chi2 values etc.) should be also presented all the fitting.

A: We understand the reviewer's concern about the accuracy of determining the refractive index, a central aspect of our work. In fact, calculating the effective refractive index in our system is particularly challenging due to the nature of the samples: transparent thin films with refractive indices close to those of the fused silica substrate. This similarity reduces the contrast in the ellipsometry signal, making the fitting process more sensitive to experimental noise. Nevertheless, the ellipsometry fittings are accurate enough to confirm the refractive index trend across the sample series. To improve reliability, we performed each fit simultaneously for five different incident angles and two polarizations. This helps constrain the model and reduce ambiguity. While individual measurements may not perfectly match the fit, the global trend across all angles is fairly well reproduced. To further support our findings, we have included root mean square error (RMSE) values for each fit in the new version of the Supporting Information (see Table R2 and Table S1). These metrics quantitatively assess the quality of the fits and confirm that the extracted refractive index values are robust.

Composite	$T_{calc.}$ (°C)	Average Δ RMSE	Average ψ RMSE
100:0	1000	24.5	3.93
90:10	1000	12.4	1.28
80:20	1000	11.5	0.963
70:30	1000	11.2	0.855
60:40	1000	9.37	0.579
50 :50	1000	8.35	0.671
33 :67	1000	5.71	1.39

Table R2: RMSE values of the ellipsometry fittings.

R2: 6) It is also necessary to give the error bars on the values as the article conclusion relies on the effect of these values.

A: We agree that error estimates are important for evaluating the reliability of experimental data. However, the parameters obtained from refractive index and composition values derived from spectroscopic fittings are obtained through numerical optimization rather than direct measurements. Therefore, standard error bars cannot be provided. Nevertheless, we can estimate a typical accuracy of ±0.02 for refractive index determination of thin films under standard conditions. We have addressed this issue in the revised version of the manuscript (see Methods).

R2: 7) Why closely matching SiO_2 nanoparticles are necessary to prepare the films?

A: Although it is not strictly necessary, using size-matched (monodisperse) nanoparticles when preparing nanocomposite films helps ensure homogeneity and transparency. Mixing nanoparticles of significantly different sizes can lead to agglomeration during film formation, resulting in inhomogeneous coatings of poor optical quality. For this reason, we selected ZGO and SiO_2 nanoparticles with similar diameters to promote uniform dispersion and minimize scattering. We have commented on this point in the amended manuscript (see page 3, line 59).

R2: 8) It is also not understood why the films were sintered at two temperatures. It is obvious that more transparency will be obtained at higher temperature than why lower temperature sintering was investigated. The advantage of sintering at 800 ºC is not clear.

A: Our goal was not to maximize transparency; rather, we sought to explore how thermal treatment conditions influence the effective refractive index of the films, a factor central to our strategy for tuning luminescence properties. Annealing at 1000 °C partially melts the SiO_2 nanoparticles, enabling them to fill the voids between the ZGO nanoparticles. This densification increases the effective refractive index at low SiO_2 content and decreases it at higher content, enabling a broad, non-monotonic tuning range. In contrast, annealing at 800 °C leaves the SiO_2 nanoparticles in the nanocomposite films intact. Consequently, films with high SiO_2 content retain their original porosity, and the effective refractive index decreases smoothly as high-index ZGO nanoparticles are replaced by low-index SiO_2. This lower-temperature treatment allows us to achieve refractive index values that cannot be reached at 1000 °C, particularly in the low-index range. Combining both annealing conditions allows for wider, more controlled modulation of the refractive index, which is essential for the tuning of the PersL properties demonstrated in this work. We have further clarified this in the revised manuscript (see page 7, line 122).

R2: 9) The error bars on strain values also necessary.

A: Error bars were obtained by propagating the standard uncertainties of the refined lattice parameter to the calculated interplanar spacings and, subsequently, to the strain values, leading to uncertainties of ~1×10^{-4}. Since the calculated strain values are on the order of 10^{-2} (e.g., 0.0198), the associated errors are at least two orders of magnitude smaller and therefore not visible in the graphs, as they fall within the symbol size. We have commented on this in the new version of the manuscript (see Methods).

R2: 10) The relation between porosity and refractive index should be also presented. This relation may also help to understand the variation of refractive index which is maximum for a particular ratio.

A: Understanding the relationship between porosity and refractive index is central to interpreting the trends observed in our nanocomposite films. In our study, we extracted the effective refractive index by fitting spectroscopic measurements using a Bruggeman effective medium approximation. This model enabled us to estimate the filling fraction (ff) of ZGO, SiO_2, and air (porosity) in the films. At 1000 °C, the partial melting of SiO_2 nanoparticles leads to the densification of the films, which reduces porosity and increases the refractive index for low SiO_2 content. As the SiO_2 content increases, the proportion of low-index material dominates and the refractive index begins to decrease. At 800 °C, the shape of the SiO_2 nanoparticles is preserved, and the porosity remains relatively high. This results in a smoother, monotonic decrease in the refractive index as the SiO_2 content increases, reaching values lower than those attainable at 1000 °C. The interplay between densification and composition explains the non-monotonic refractive index behavior observed at 1000 °C and the monotonic trend at 800 °C. We have clarified this relationship and the use of the Bruggeman model in the revised manuscript (see page 7, line 108 and page 7, line 118).

R2: 11) The increment in luminescence and product of decay rate also need to be explained. Why one value increase by a factor of 1.8 and another by 1.21. How these values align trend wise. The mechanism should be explained.

A: Enhancements in luminescence intensity and radiative decay rate, corrected for the amount of active material (M_{ZGO}), follow similar trends. However, they are not expected to match exactly, as emission intensity is also affected by film morphology and light scattering. These factors may influence light incoupling and outcoupling. As the referee points out, the 10% SiO_2 sample annealed at 1000 °C exhibits a 1.8-fold increase in luminescence, while the calculated increase in the product of the radiative decay rate and the amount of active material ($\Gamma_{rad}\cdot M_{ZGO}$) is ~1.21-fold. Additionally, PLQY and decay measurements (see Figure S7 in the Supporting Information) show a 1.41-fold enhancement for the same sample, which lies between the calculated and observed values. We attribute this discrepancy to the difference in the scattering properties of the nanocomposite compared to the reference ZGO film. As previously mentioned in this response letter, scattering affects the incoupling and outcoupling of light. This can lead to more effective excitation and improved light extraction. We have addressed this issue in the revised manuscript (see page 9, line 159).

R2: 12) Trend of values (maximum at particular value while another decrease with SiO_2 ratio) is also need to be explained with a better mechanism.

A: As previously discussed, the luminescence and persistent luminescence intensities exhibit non-monotonic behavior with respect to the SiO_2 content, especially for samples annealed at 1000 °C. This trend is primarily governed by the interplay between changes in the radiative rate due to modifications in the effective refractive index and the amount of active material in the

film composition. At low SiO_2 content, the partial melting of SiO_2 nanoparticles results in denser films and an increased effective refractive index. This enhances the radiative decay rate, improving light generation and luminescence intensity. However, as the SiO_2 increases further, the luminescence intensity decreases due to the reduction in the amount of active material in the nanocomposite films. This competing behavior is well captured by the product $\Gamma_{rad} \cdot M_{ZGO}$, which also peaks at intermediate SiO_2 content values. We have clarified this point further in the new version of the manuscript, following the reviewer's advice (see page 11, line 179).

R2: 13) The role of trap centers is the most important factor for persistent luminescence and therefore should be presented for all samples with detailed analysis. In place of having results for 800 ºC, it may be more useful to have defects related analysis in details.

A: We agree that the trap landscape plays a central role in determining afterglow behavior. In our study, we examined this phenomenon using TL measurements. As the TL data show (see Fig. S8 of the Supporting Information), the inclusion of SiO_2 has a minimal impact on the TL glow curves. This suggests that the distribution of trap depths in ZGO remains largely unaffected by the presence of SiO_2 nanoparticles in the nanocomposite films. Consequently, the persistent luminescence decay kinetics remain nearly unchanged (see Fig. R2a). We have commented on this in the amended version of the manuscript (see page 12, line 201).

R2: 14) Whether the TL intensity was normalised to the PL intensity as samples having higher luminescence efficiency will give higher intensity of TL glow peaks as well.

A: We agree with the reviewer that samples with stronger afterglow also have stronger TL signals because both phenomena are governed by trap population dynamics. However, the limitations of the experimental setup we used for this part of the characterization do not allow for a fair comparison of absolute TL intensities. For this reason, we chose to analyze TL glow curves that were normalized in intensity. We have clarified this point in the revised manuscript (see page 17, line 307).

R2: 15) Persistent luminescence should be presented, in terms of remaining intensity after particular time for all samples. The initial Persistent luminescence intensity is not as important as its variation with time. Initial intensity may be high but doesn't have practical applications if decays faster.

A: In our study, we focused on analyzing the integrated PersL signal during the afterglow period. As the time-dependent PersL intensity measurements displayed in Fig. R2a (new Figure S8b), all samples exhibit very similar afterglow decay profiles regardless of the SiO_2 content or processing conditions. This indicates that the trap distribution and release dynamics remain largely unchanged across the different nanocomposite films, as discussed in our response letter. Consequently, the afterglow intensity enhancement factor remains nearly constant throughout the afterglow period. We have further clarified this point in the revised manuscript (see page 11, line 184 and new Figure S8b).

R2: 16) The claim of "Precisely tuning of afterglow and kinetics" is not supported with the available data and mechanism.

A: We respectfully disagree with the reviewer. We are convinced that the data and analysis presented in the manuscript support the claim of precise tuning of afterglow intensity and kinetics. The tuning of afterglow intensity is clearly demonstrated by the systematic variation of the effective refractive index. This is supported by experimental measurements and theoretical modeling. Regarding the kinetics, we used a validated rate equation model to fit the luminescence and persistent luminescence charging curves. The results of these fits revealed that the charging rate (p_1) varies with SiO_2 content and annealing temperature while the trap

release rate (p_2) remains constant. This suggests that strain induced by SiO_2 inclusion affects charging dynamics without altering trap depth distribution. Based on these findings, we demonstrate that our nanocomposite strategy enables independent modulation of the intensity and kinetics of persistent luminescence without altering the ZGO composition, representing a landmark in the field.
